# Supplementary material for: Reconfigurable halide perovskite nanocrystal memristors for neuromorphic computing
Source: Nat Commun. 2022 Apr 19;13:2074. doi: 10.1038/s41467-022-29727-1 (PMC9018677; doi:10.1038/s41467-022-29727-1)
Supplement: Supplementary file 1 — Supplementary Information [file 41467_2022_29727_MOESM1_ESM.pdf]

Supplementary Information

Reconfigurable Halide Perovskite Nanocrystal Memristors for Neuromorphic Computing

John et al.

Supplementary Note 1: Synthesis and characterization of halide perovskite nanocrystals

Synthesis of DDAB-capped $CsPbBr_3$ nanocrystals.

$PbBr_2$ (55 mg, 0.15 mmol) and DDAB (61 mg, 0.132 mmol) were added to 4 mL triethylbenzene. With vigorous magnetic stirring, the mixture was heated to 180°C until it became clear, and Cs-Diisooctylphosphinic acid (DOPA) precursor solution of 0.4 mL was swiftly injected to this. After 10 secs, the reaction mixture was cooled down to room temperature with a water/ice bath.

Purification of DDAB-capped $CsPbBr_3$ nanocrystals.

0.5 mL of toluene and 1 mL acetone were added to 0.5 mL of the crude solution, followed by centrifugation for 3 min at 10 krpm. The supernatant was discarded, and the precipitate was redispersed in 0.5 mL of cyclohexane. All obtained solution of $CsPbBr_3$ nanocrystals was centrifuged for 20 min at 12.1 krpm. The resulting supernatant was filtered with a PTFE-syringe filter (pore size 0.2μm) and used for device fabrication.

Synthesis of OGB-capped $CsPbBr_3$ nanocrystals.

Lead acetate (49.3 mg, 0.13 mmol), cesium carbonate (10.6 mg, 0.0325 mmol), hexanoic acid (0.231 mL, 1.85 mmol) and mesitylene (5 mL) were loaded in a 25mL three-necked-flask. With vigorous magnetic stirring, the mixture was heated to 90°C. After reaching the reaction temperature, Oleylguanidinium Bromide (252.5 mg, 0.65 mmol) solution in 1 mL mesitylene was injected using a 3-mL syringe with a 1.6x 40mm needle. After 10s the reaction mixture was cooled down to RT with an ice bath.

Purification of OGB-capped $CsPbBr_3$ nanocrystals.

0.3 mL of butyl acetate and 0.15 mL methyl acetate were added to 0.5 mL of the crude solution, followed by the centrifugation for 4 min at 11 krpm. The precipitate was discarded, and 0.15 mL methyl acetate was added to the resulting supernatant, followed by the centrifugation for 4 min at 11 krpm. The precipitate was redispersed in 0.15 mL of cyclohexane. All obtained solution of $CsPbBr_3$ nanocrystals was filtered with a PTFE-syringe filter (pore size 0.2μm) and used for device fabrication.

Rationale for ligand selection.

Didodecyldimethylammonium bromide molecule (DDAB) is a quaternary ammonium salt with two methyl groups and two carbon chains consisting of 12 carbon atoms, whereas oleylguanidinium bromide (OGB) is a guanidinium salt with one unsaturated chain containing 18 carbons. One of the most important advantages of OGB is the ability of the guanidinium group to create multiple hydrogen bonds with halides on the NC surface that can result in the better binding of such ligands to the surface of lead halide perovskite NCs.

The second main advantage of OGB molecule is that the longer carbon chain improves colloidal stability of the NC solution.

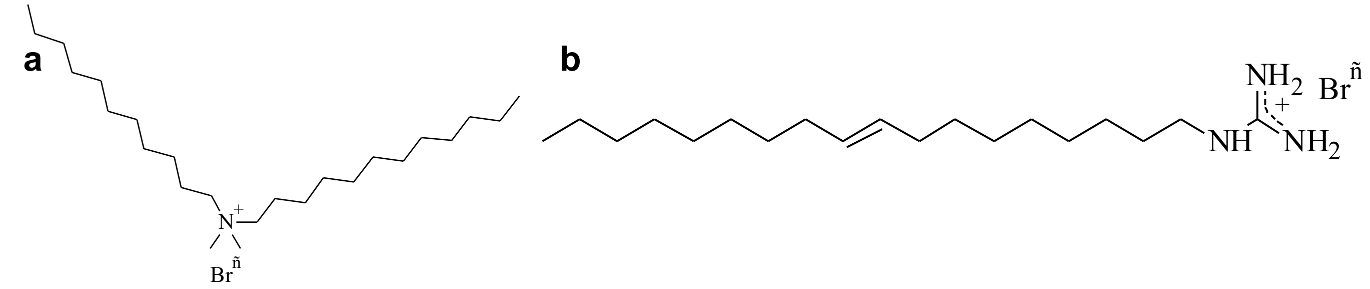

Supplementary Fig. 1. Ligand structure. a Structure of the DDAB (didodecylammonium bromide) and **b** OGB (oleylguanidinium bromide) ligand.

It is known that commonly used ligands in the synthesis of lead halide perovskite NCs (oleic acid and oleylamine) loosely bind to the NC surface. This leads to the loss of colloidal stability and structural integrity of perovskite NCs. To overcome instability issues, a new generation of organic ligands with various head groups should be developed and used. $CsPbBr_3$ NCs with quaternary ammonium and novel guanidinium-based long-chain ligands on the surface seem promising in this regard.

The attachment of ligands to the cesium halide terminated surface of perovskite NCs can be imagined as a replacement of some cesium ions by the head groups of the cationic ligands such as DDAB or OGB. It has already been shown that DDAB ligands improve the chemical stability of perovskite NCs and allow their purification preserving high photoluminescence quantum yield[1]. At the same time, we developed a synthesis of OGB-capped $CsPbBr_3$ NCs because we assume that the guanidinium binding group in OGB molecule has a higher ability to create multiple hydrogen bonds with halides on the NC surface in comparison to DDAB molecule that can result in stronger binding to the $CsPbBr_3$ NCs.

Another difference between DDAB and OGB ligands is in their packing density on the surface of perovskite NCs. Carbon chains of DDAB are bulky and stand out which makes their fitting on the NC surface complicated. On the contrary, OGB molecule has only one oleyl chain with a double bond that, most probably, will allow packing of more ligands on the surface of perovskite NCs. DDAB ligand has shorter carbon chains what makes them more useful in applications where charge transfer is important, such as LED devices. However, OGB has a longer carbon chain creating a bigger distance between NCs and making charge transfer harder. All these features described above can strongly impact the performance of devices based on DDAB or OGB-capped $CsPbBr_3$ NCs.

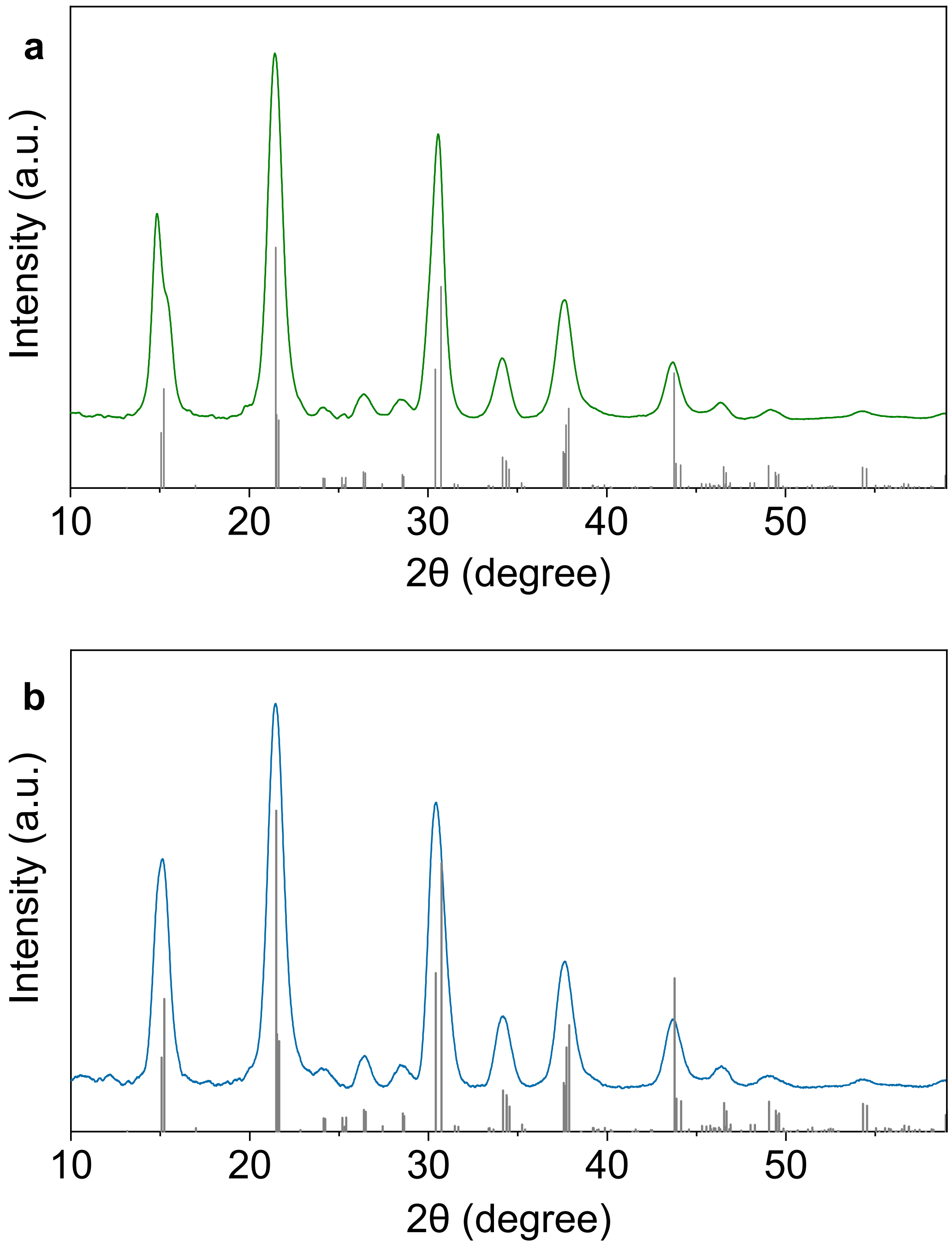

Supplementary Fig. 2. X-ray diffraction (XRD) of **a** DDAB and **b** OGB-capped $CsPbBr_3$ NC films.

Supplementary Note 2: Working mechanism, IV characteristics, Thin film characterization

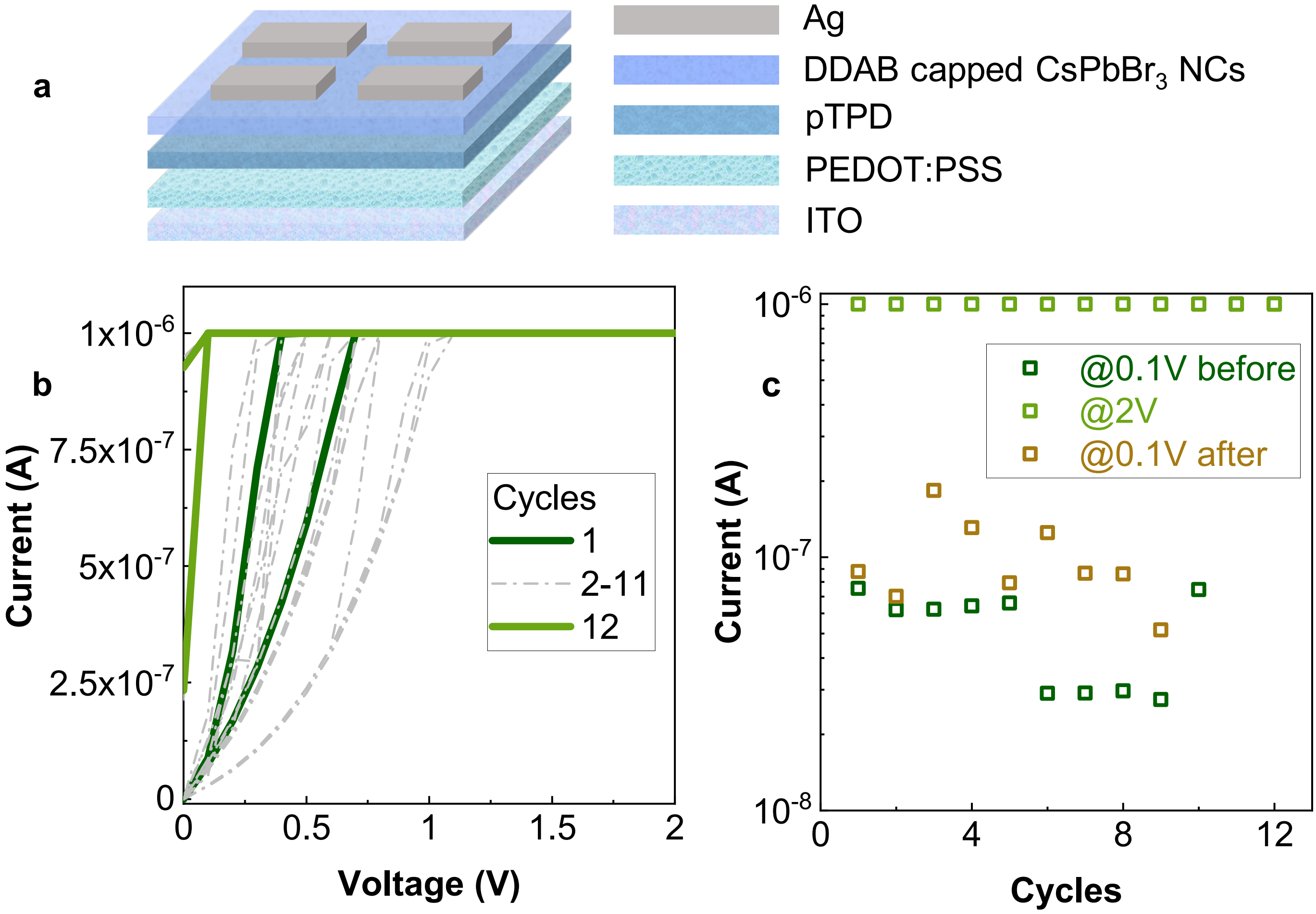

Supplementary Fig. 3. DDAB capped $CsPbBr_3$ NC memristor. a Device structure. **b** Representative IV characteristics and **c** Endurance of volatile diffusive threshold switching.

Diffusive behaviour: The volatile threshold switching behaviour can be attributed to the redistribution of Ag^+ and Br^- ions under an applied electric field, and their back-diffusion upon removing power (Supplementary Fig. 4). The soft lattice of the halide perovskite matrix has been observed to enable Ag^+ migration with an activation energy ~ 0.15 eV[2]. Interestingly, migration of halide ions and their vacancies within the perovskite matrix also occur at similar energies ~ 0.10 - 0.25 eV[3–5], making it difficult to pinpoint a singular operation mechanism. We hypothesize that during the SET process, Ag atoms are ionized to Ag^+ and forms a percolation path through the device structure. Electrons from the grounded electrode oxidize Ag^+ to form weak Ag filaments. Parallelly, Br^- ions get attracted towards the positively charged electrode and a weak filament composed of vacancies (V_{Br}) is formed. Both these factors increase the device conductance from a high resistance state (HRS) to a temporary low resistance state (LRS). Upon removing the electric field, the low activation energy of the ions causes them to diffuse back spontaneously, breaking the percolation path and leading to volatile memory characteristics a.k.a. short-term plasticity. The low compliance current (I_{cc}) of 1 μA ensures that the electrochemical reactions are well regulated, and the percolation pathways formed are weak enough to allow these diffusive dynamics.

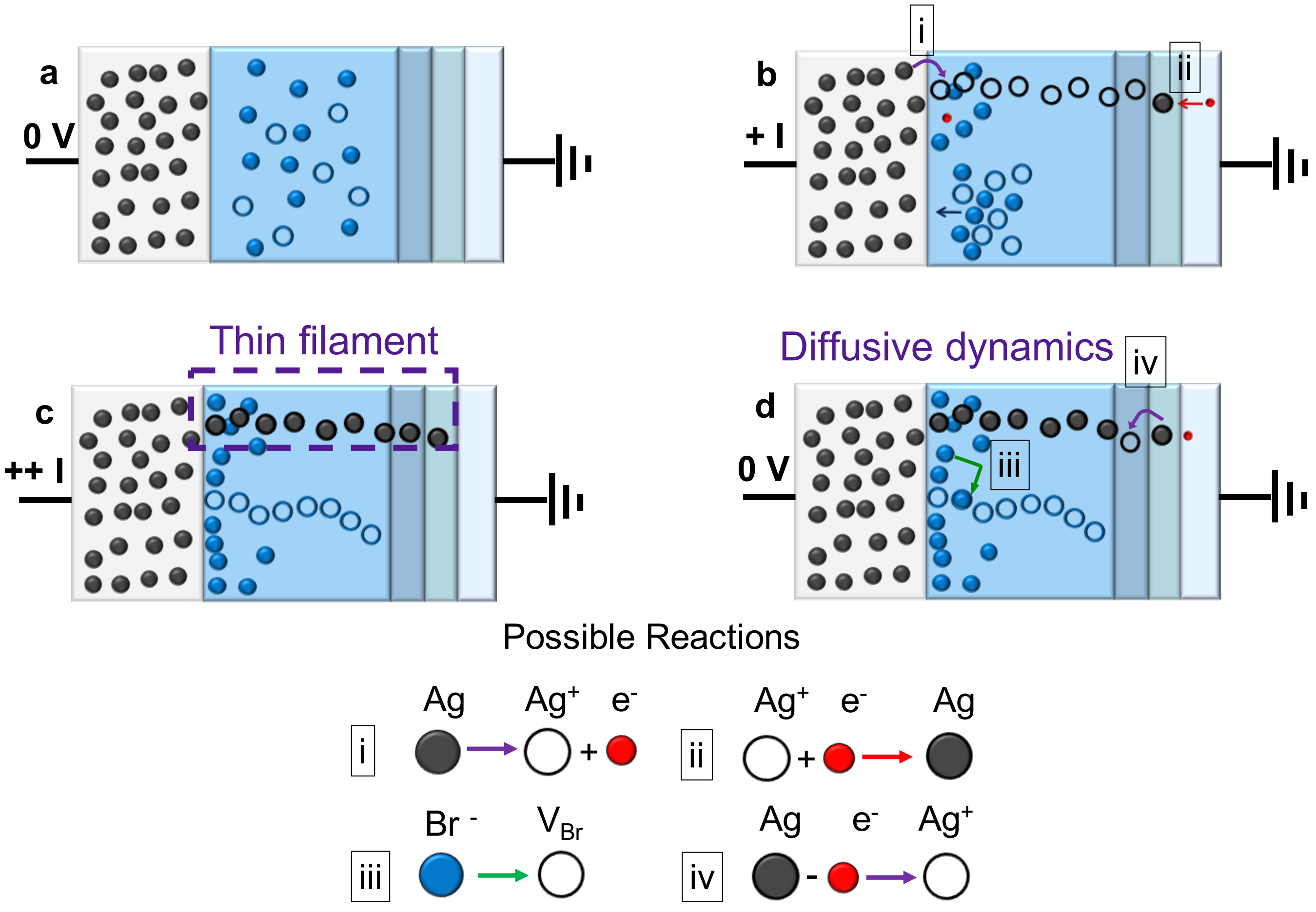

Supplementary Fig. 4. Proposed volatile diffusive switching mechanism. a-d illustrate the various stages of filament formation and rupture. i-iv indicate the possible reactions happening within the device.

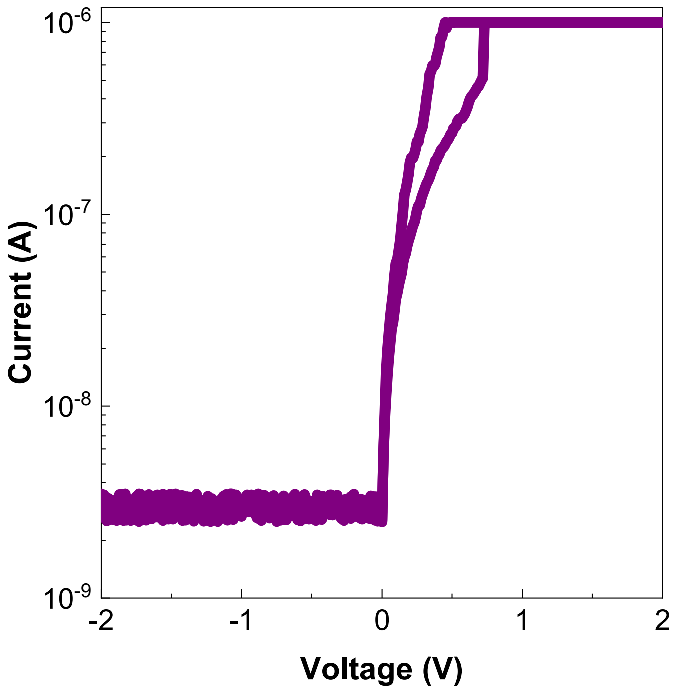

Both DDAB and OGB devices exhibit a unidirectional DC threshold switching behaviour (Supplementary Fig. 5) with no switching occurring under reverse bias. A representative IV characteristic of OGB-capped $CsPbBr_3$ memristor is shown here.

Supplementary Fig. 5. Unidirectional Threshold Switching. Bidirectional IV sweep of OGB-capped $CsPbBr_3$ memristor in the volatile mode.

Drift behaviour: Upon increasing the I_{cc} to 1mA (3 orders of magnitude higher than that used for volatile threshold switching), permanent and thicker conductive filamentary pathways are possibly formed within the device as illustrated in Supplementary Fig. 6. This increases the device conductance from a high resistance state (HRS) to a permanent and much lower low resistance state (LRS). Electrochemical reactions are triggered to a higher extent and hence, the switching dynamics is now dominated by the drift kinetics of the mobile ion species Ag^+ and Br^-, rather than diffusion. Hence upon removing the electric field, the conductive filaments remain largely unaffected, and the devices retain their LRS and portray long-term plasticity. Application of voltage sweeps, or pulses of opposite polarity causes rupture of these filaments, and the devices are reset to their HRS. For DDAB-capped $CsPbBr_3$ NCs, the devices transition to a non-erasable non-volatile state within ~ 50 cycles, indicating formation of very thick filaments (Supplementary Fig. 7). On the other hand, the OGB-capped $CsPbBr_3$ NCs display a record-high non-volatile endurance of 5655 cycles (Fig. 3b) and retention of 10^5 seconds (Supplementary Fig. 8), pointing to better regulation of the filament formation and rupture kinetics.

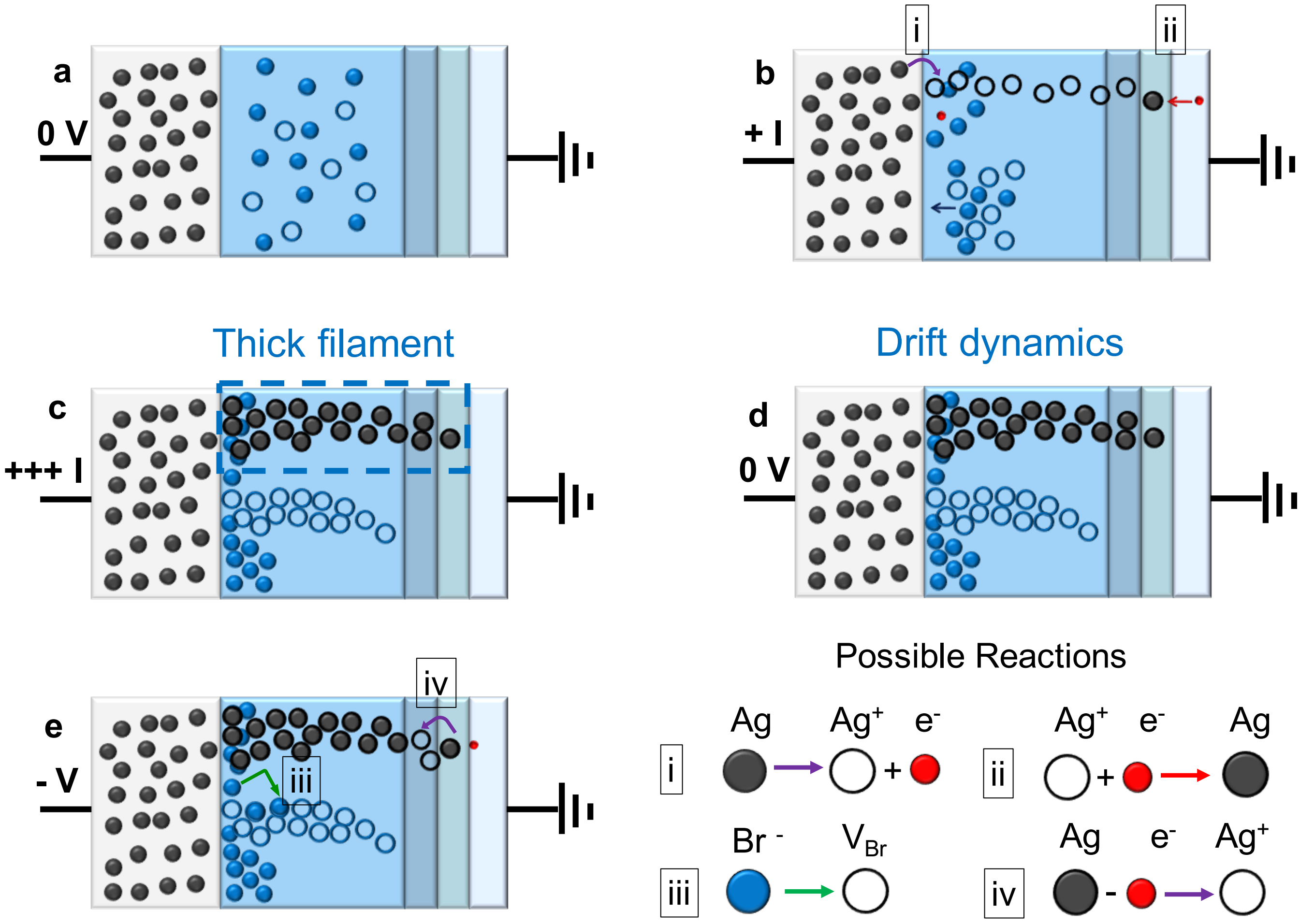

Supplementary Fig. 6. Proposed non-volatile drift switching mechanism. a-e illustrate the various stages of filament formation and rupture. i-iv indicate the possible reactions happening within the device.

It is important to note that the abrupt jump in conductance seen in the IV characteristics (Fig. 3) at both low compliance current (I_{cc}) of 1μA and high I_{cc} of 1mA points to conductive filament (CF) formation in both the volatile and non-volatile modes, albeit with contrasting number or size, in alignment with literature[6–8].

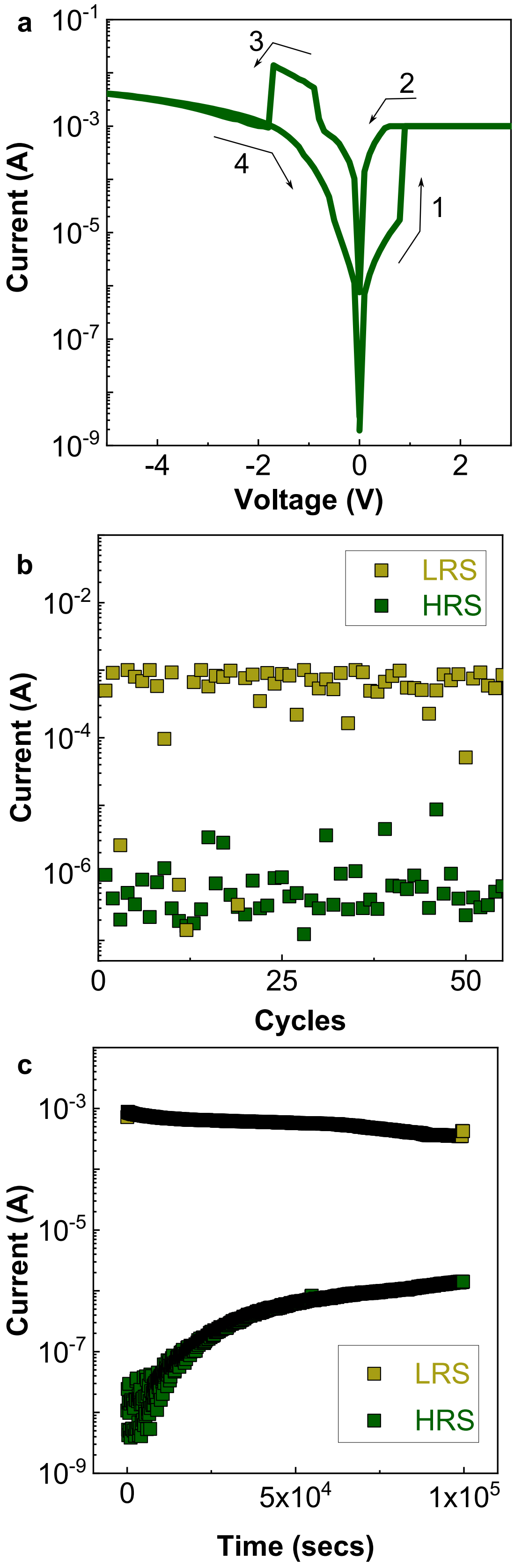

Supplementary Fig. 7. Non-volatile drift switching of DDAB-capped $CsPbBr_3$ NC memristors. a Representative IV characteristics. **b** Endurance. **c** Retention.

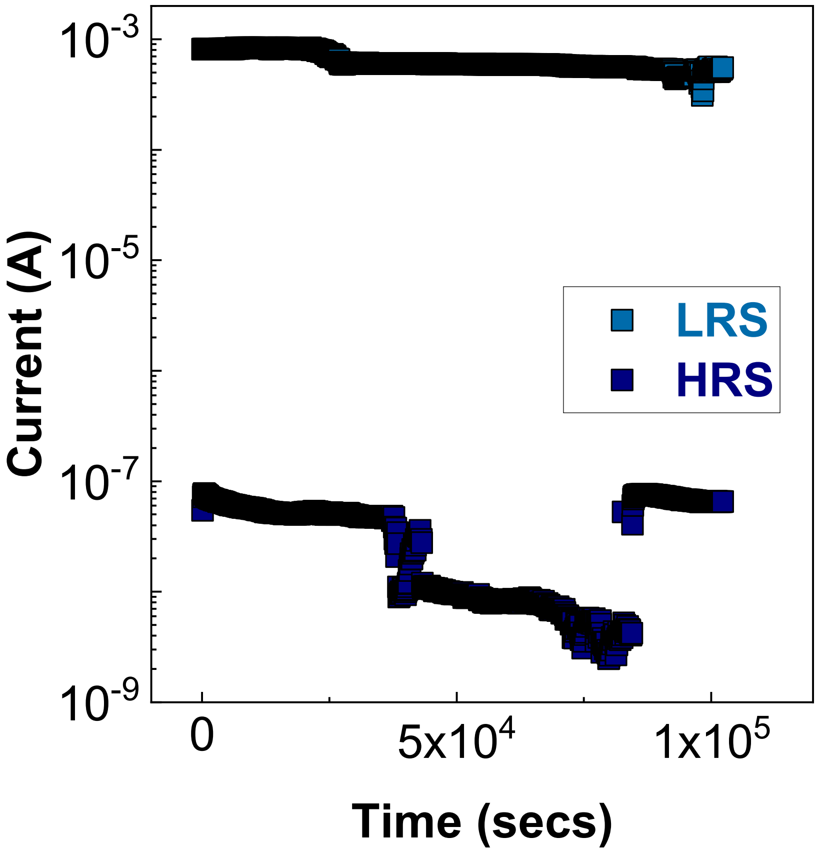

Supplementary Fig. 8. Non-volatile drift switching of OGB-capped $CsPbBr_3$ NC memristors. Figure shows the retention performance.

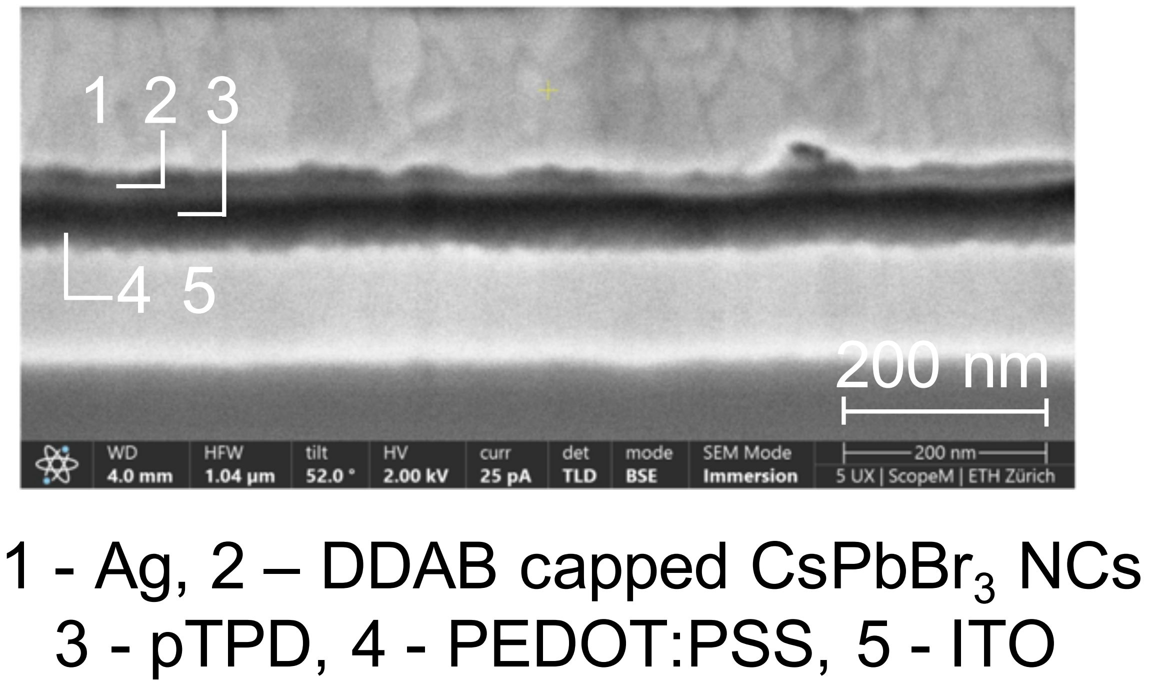

Supplementary Fig. 9. Scanning electron microscope (SEM) images of the device cross section of DDAB-capped $CsPbBr_3$ NC memristors.

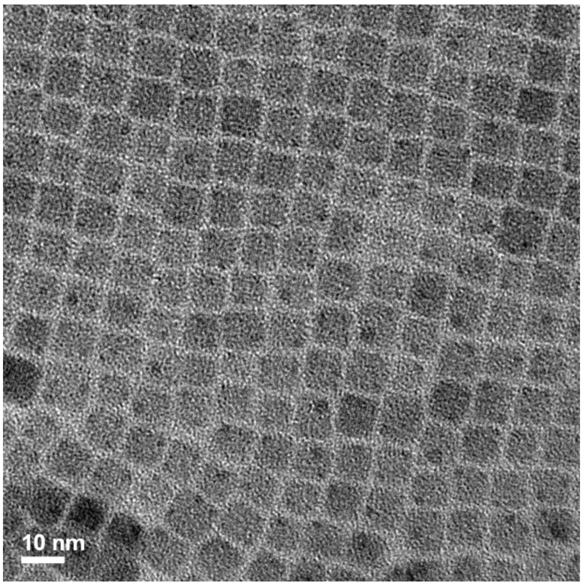

Supplementary Fig. 10. Transmission electron microscope (TEM) images of DDAB-capped $CsPbBr_3$ NCs.

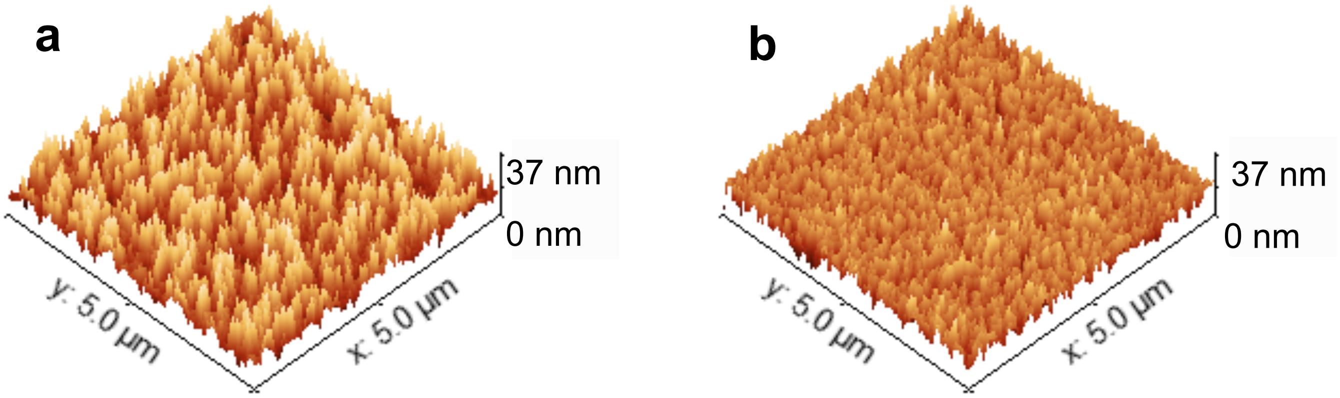

Supplementary Fig. 11. Atomic force microscope images of **a** DDAB and **b** OGB-capped $CsPbBr_3$ NC films. Statistical analysis reveals root mean square (rms) roughness values of 5 and 3.5 nm respectively.

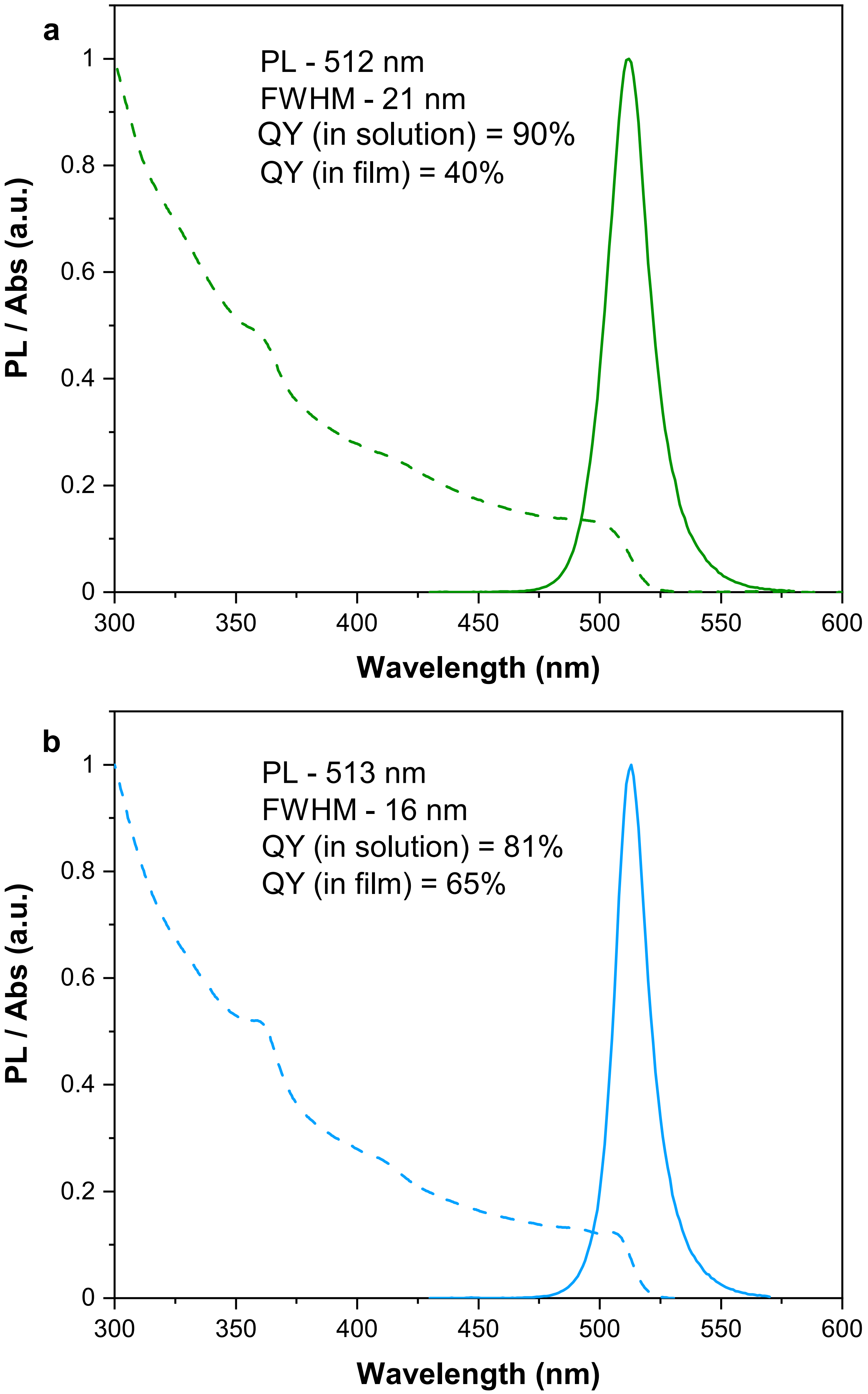

Supplementary Fig. 12. Photoluminescence and absorption spectroscopy of **a** DDAB and **b** OGB-capped $CsPbBr_3$ NCs in solution and thin films.

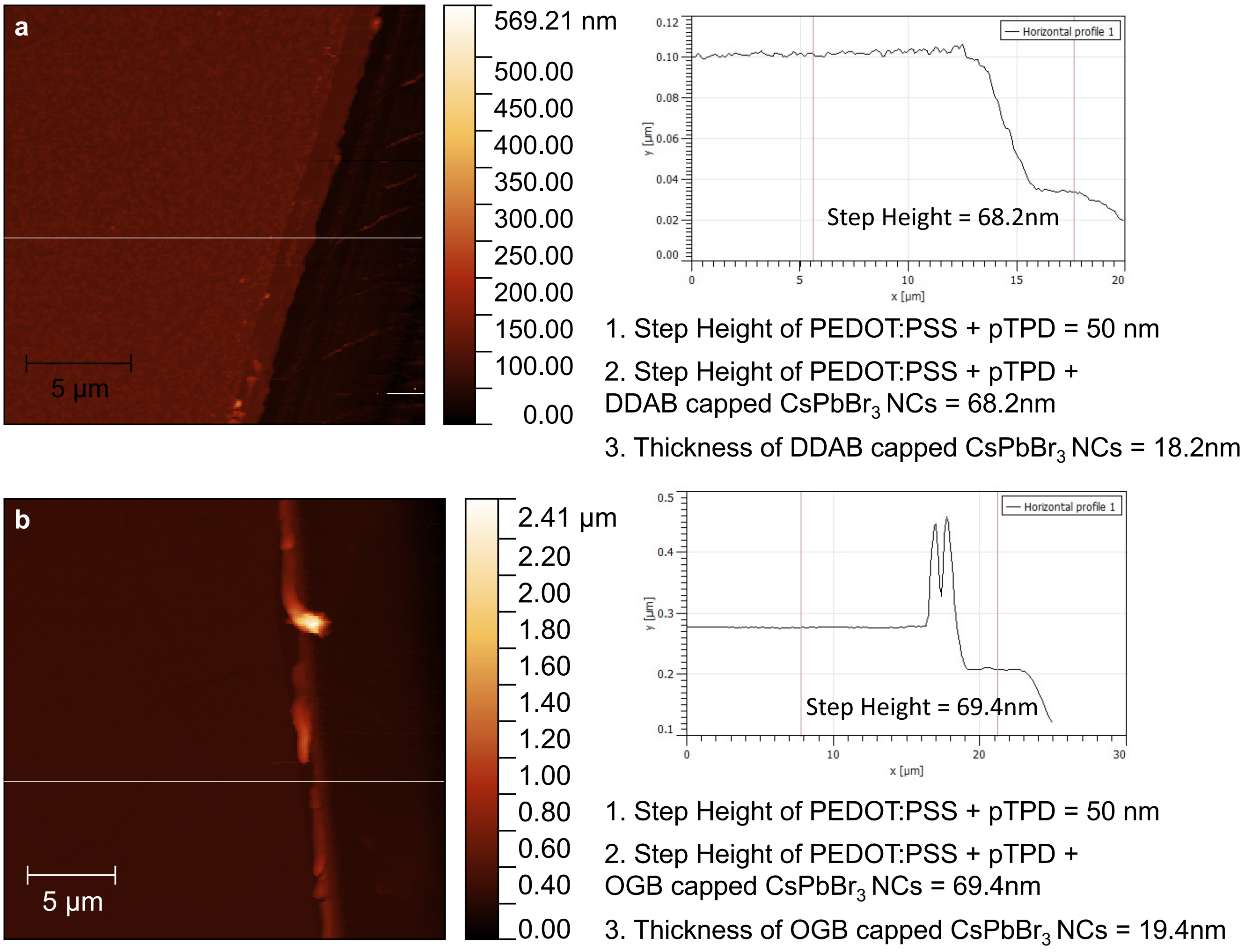

Supplementary Fig. 13. Thickness determination. AFM scan area of PEDOT:PSS + pTPD + $CsPbBr_3$ NCs (left). Step height profile (right) of the white line shown on the left. Thickness of the $CsPbBr_3$ NC thin film is calculated in a subtractive manner as indicated by the text in the figure. **a** and **b** shows the result of DDAB and OGB-capped $CsPbBr_3$ NCs respectively.

Investigation with Au as the active electrode

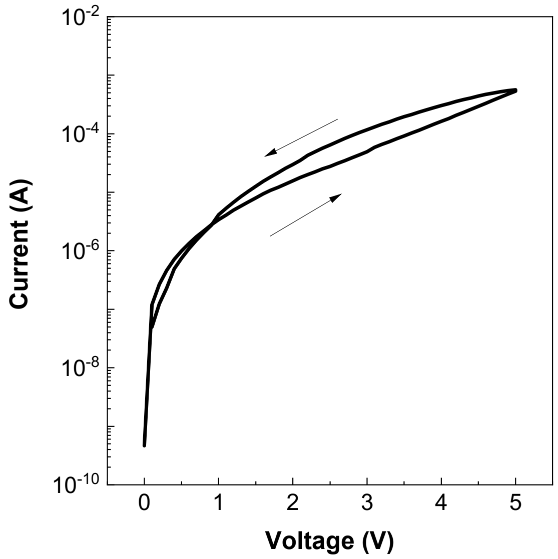

Supplementary Fig. 14. Effect of Au on the switching characteristics of OGB-capped perovskite NC memristors.

This experiment indicates that the resistive switching behaviour in our devices is solely due to Ag filaments. However, it has to be noted that the nanoscopic electrochemical environment induced by the halide migration during the voltage application could create more favourable pathways for the drift and diffusion of Ag^+ ions. This has been observed previously in solar cell configurations even across organic transport layers[9–11]. Hence, it becomes challenging to completely rule out the effect Br^- migration as a possible contributing factor to the resistive switching.

Role of PEDOT:PSS and pTPD interlayers

The PEDOT:PSS + pTPD interlayers allow better perovskite NC thin film formation. The coating of $CsPbBr_3$ NC thin film directly on ITO results in clusters of NCs with poor adhesion. On PEDOT:PSS, the films are better when compared to ITO. But the presence of large crevices once again indicate poor film formation of the NCs. Devices built with this configuration (ITO/ PEDOT:PSS/ $CsPbBr_3$ NC thin film/Ag) do not give reliable resistive switching and multiple devices are short (data not shown). On the other hand, $CsPbBr_3$ NC thin film formation is best when coated on PEDOT:PSS + pTPD interlayers as evident from the AFM images (Supplementary Fig. 15). This is in alignment with similar observations on halide perovskite solar cells[12].

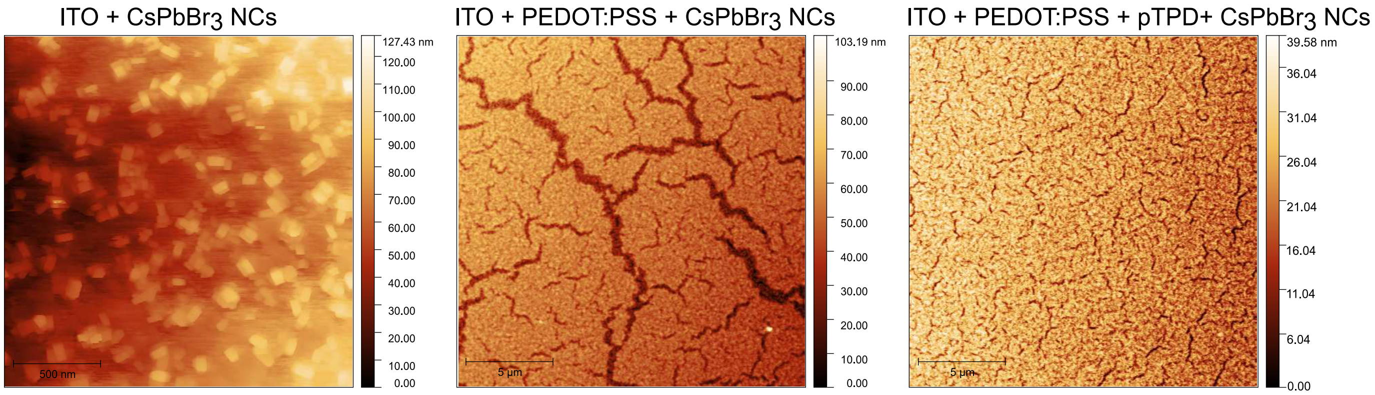

Supplementary Fig. 15. Role of PEDOT:PSS and pTPD interlayers. AFM images of $CsPbBr_3$ NC thin film on ITO (scan area = 2 µm × 2 µm), ITO + PEDOT:PSS (scan area = 20 µm × 20 µm) and ITO + PEDOT:PSS + pTPD interlayers (scan area = 20 µm × 20 µm).

IV measurements on ITO/PEDOT:PSS/Ag and ITO/PEDOT:PSS + pTPD/Ag devices showed no memristive behaviour, proving that these interlayers do not contribute to the resistive switching behaviour (Supplementary Fig. 16). This reiterates the importance of the perovskite NC thin film as an active matrix for reliable Ag filament formation and rupture, leading to realization of robust memristors.

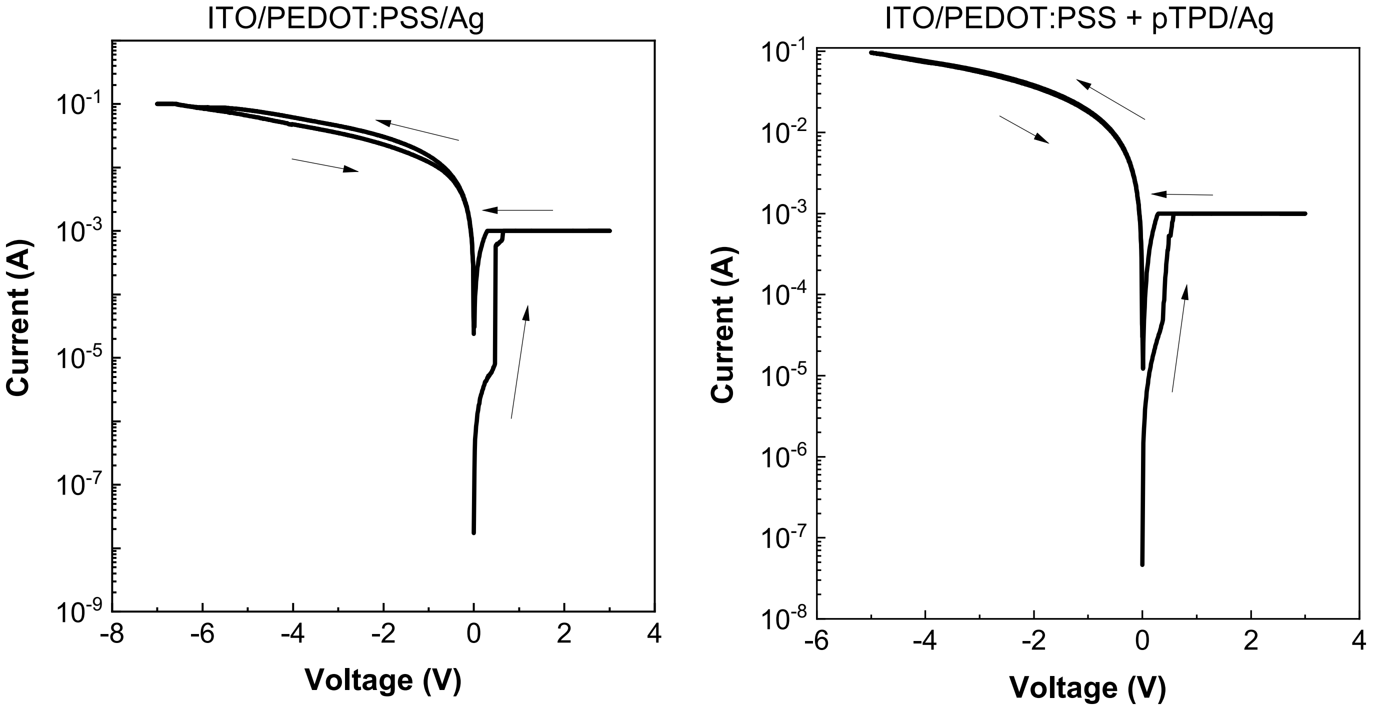

Supplementary Fig. 16. IV of PEDOT:PSS and pTPD-only devices. IV sweep of ITO/PEDOT:PSS/Ag and ITO/PEDOT:PSS + pPTD/Ag devices.

Secondary ion mass spectrometry (SIMS) results:

The SIMS scans reveal a clear difference in the ^{107}Ag cross section profile when comparing an ON and OFF device. In both cases, the depth profiles highlight a severe intermixing of ^{107}Ag induced by the sputtering O_2^+ ion beam. The origin of the intermixing is most likely ballistic[13] and electrically-driven[14].

When isolating the effect of the intermixing by normalizing the ^{107}Ag with respect to a referenced OFF device, the ^{107}Ag profile of the ON device displays a considerable difference (Supplementary Fig. 17). The Ag pads deposited on top of the surface seem to be Ag depleted (i.e., a lower amount of Ag), which is confirmed by microscope observation. It must be noted that the first few nanometers of the depth profiling are affected by surface contaminations and can be considered as a transient region. The interesting section is located at the interface between the halide perovskite and the organic layers (near the top red line shown in the plots, also denoted by the circle), where an increase of the ^{107}Ag count is observed.

Although, the intermixing mentioned above still affects the ^{107}Ag profile, it is clearly seen by the depth profiles, that there is ^{107}Ag present in the structure due to the operation of the device. Although the ^{107}Ag cannot be presently localized precisely in the structure, a qualitative comparison of the Ag profiles between the ON and OFF states proves formation of Ag conductive filaments upon biasing, validating our proposed memristive switching mechanism.

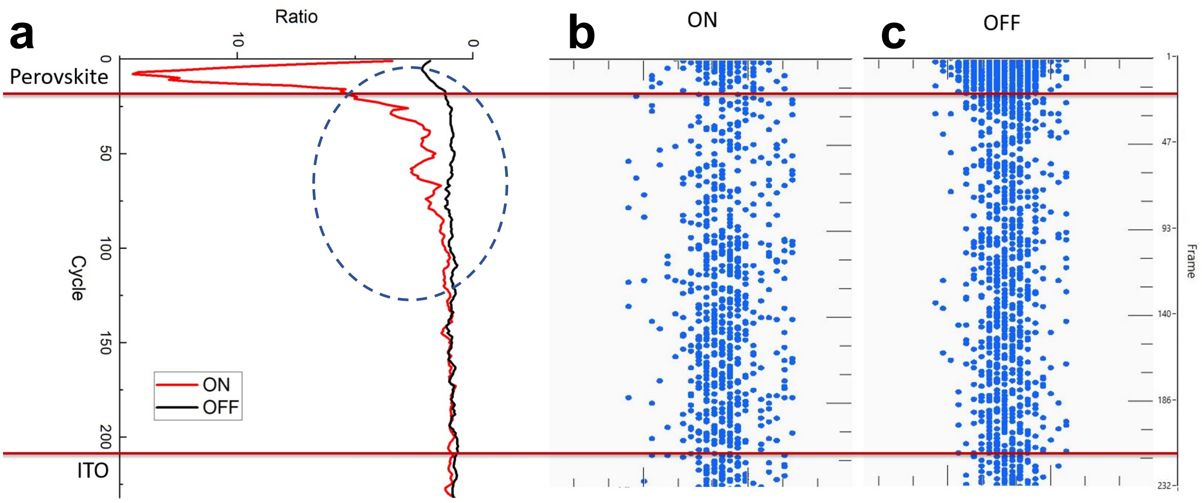

Supplementary Fig. 17. Secondary ion mass spectrometry (SIMS) results. a Ratio of an OFF and ON device with respect to the average ^{107}Ag counts per cycle normalized to a referenced OFF device, i.e., OFF/ON. 2D representation of the ^{107}Ag located in the cross section of an **b** ON and **c** OFF device.

Activation Energy for Ag^+ migration:

We begin by replotting the I–V curves on a double-log scale. The devices exhibit an Ohmic contact behaviour with a linear slope of 1 in the LRS (Supplementary Fig. 18a), supporting the hypothesized mechanism of Ag CF formation and rupture[15]. In the HRS, multiple slope features corresponding to various charge transport behaviour ($I \propto V^n$) are observed across all compositions (Supplementary Fig. 18b), pointing to trap-controlled space-charge-limited currents (SCLC) due to trapping and detrapping of injected electrons by the inherent defects in the perovskite matrix[16]. The exact dynamics in the HRS is still unclear due to hidden contributions of halide ion migration in perovskites[17,18]. To calculate the activation energy, we investigate the variation in the HRS in the voltage range 0.01-0.1V as a function of temperature using the Arrhenius equation $R = R_0 e^{-E_a/k_B T}$ (Supplementary Fig. 18c). The extracted value of 0.208eV matches values reported in literature for Ag^+ migration[2], supporting the hypothesized mechanism of Ag CF formation and rupture.

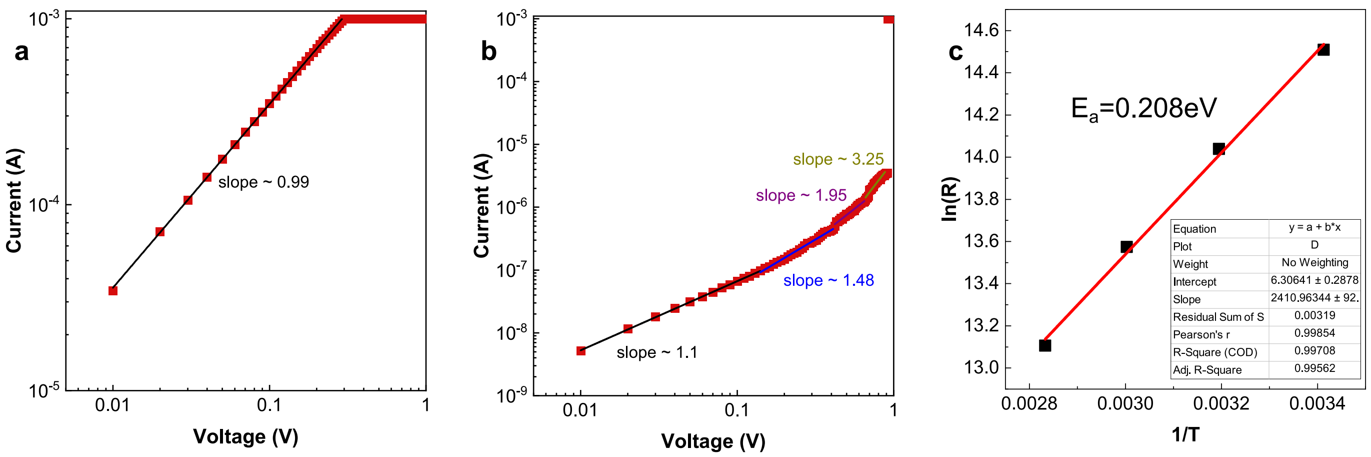

Supplementary Fig. 18. Activation energy calculation. Double log I–V curves in the positive voltage sweeping region of the memristors provide insights on the charge transport mechanism in the **a** LRS and **b**

HRS. **c** shows the calculation of the activation energy by studying the variation in the HRS as a function of temperature.

Dependence on NC layer thickness, size and dispersity

Supplementary Fig. 19 shows the device characteristics as a function of the nanocrystal layer thickness. All devices exhibit very similar characteristics with an on-off ratio $\geq 10^3$, similar set and reset voltages. Statistical analysis of the on-off ratios also reveal independence from the nanocrystal layer thickness.

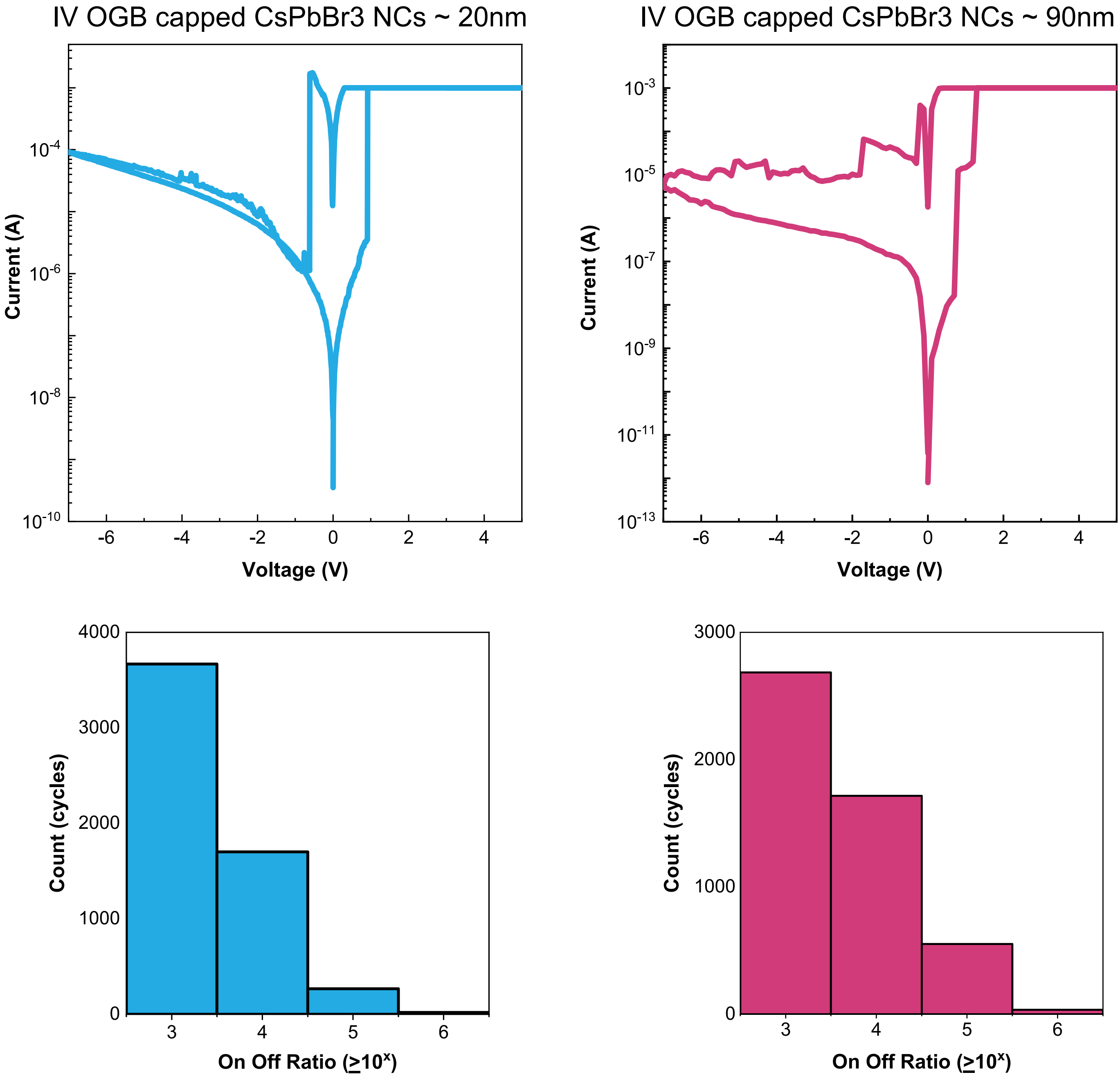

Supplementary Fig. 19. Effect of NC layer thickness. IV characteristics (top) and statistical analysis of the distribution of on-off ratios during endurance testing (bottom) of 20nm and 90nm OGB-capped $CsPbBr_3$ NC memristors.

To study the effects of NC size and dispersity, 3 solutions of OGB-capped NCs with an average size of 13, 9 and 7nm were prepared. Since NCs have a slightly elongated shape in one direction, it is more convenient to refer to the aspect ratio of the NCs. The corresponding aspect ratios were: 1.47 ± 0.23, 1.26 ± 0.36 and 1.57 ± 0.25 respectively. The DDAB-capped NCs had an average size of 9 nm with an aspect ratio of 1.09 ± 0.1. The size, aspect ratio and monodispersity was confirmed via TEM (not shown here) and photoluminescence measurements (Supplementary Fig. 20). For all colloids, we observed a narrow FWHM of PL spectra (~18 nm for all OGB samples and ~20 nm for DDAB samples) that confirms their high monodispersity.

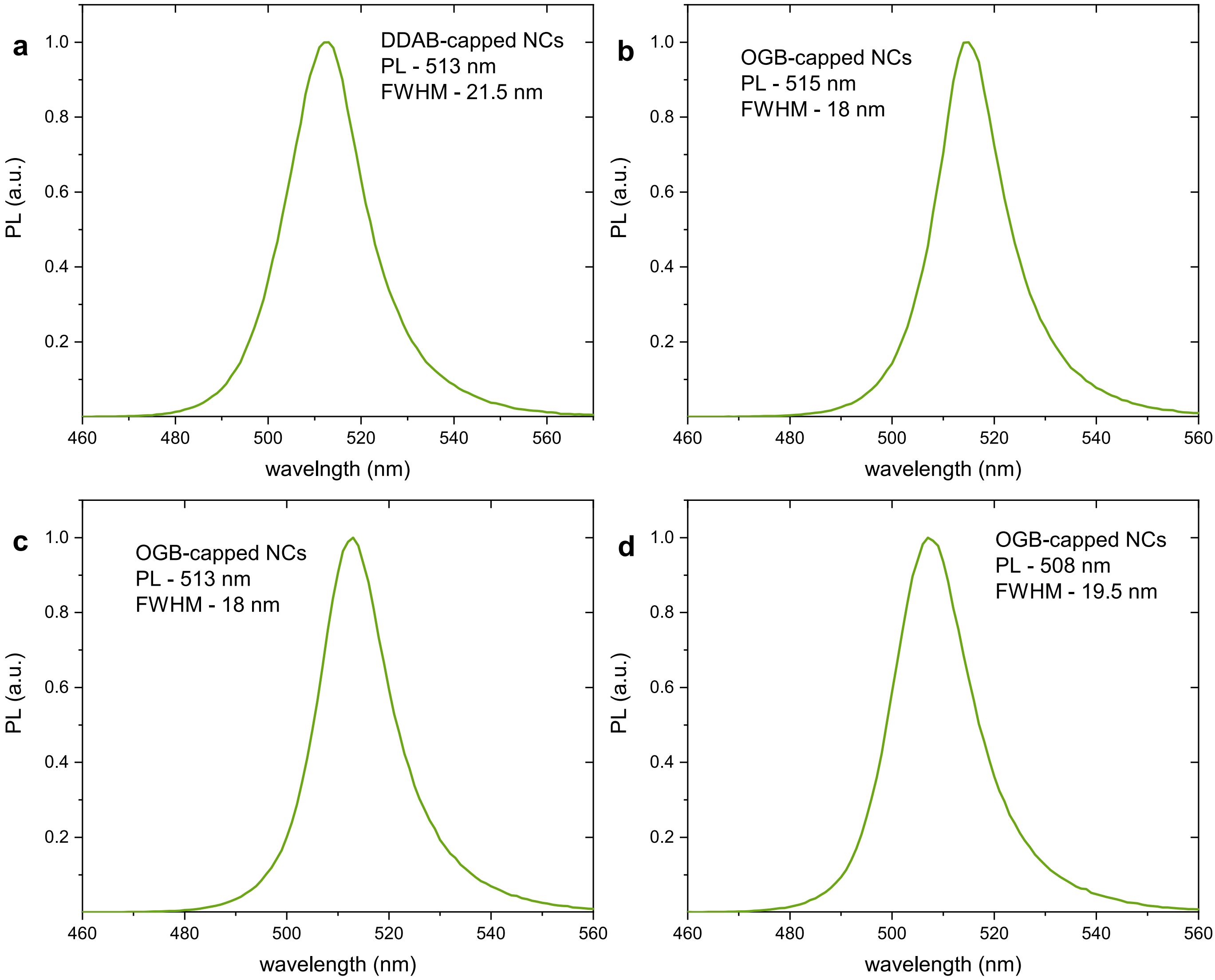

Supplementary Fig. 20. Photoluminescence spectroscopy of **a** DDAB and **b-d** OGB-capped $CsPbBr_3$ NCs of various sizes and aspect ratios in solution.

Devices fabricated with each of the solutions for comparison exhibit very similar characteristics with an on-off ratio $\geq 10^3$, similar set and reset voltages. Statistics derived from extensive measurements also do not show any trend with the nanocrystal size (Supplementary Fig. 21). While the exact mechanism is still a matter of ongoing research, the best results in terms of yield of devices with an on-off ratio $\geq 10^3$ and

endurance of > 4000 cycles in the non-volatile mode is obtained with NCs of size 9 nm with an aspect ratio of 1.26 ± 0.36.

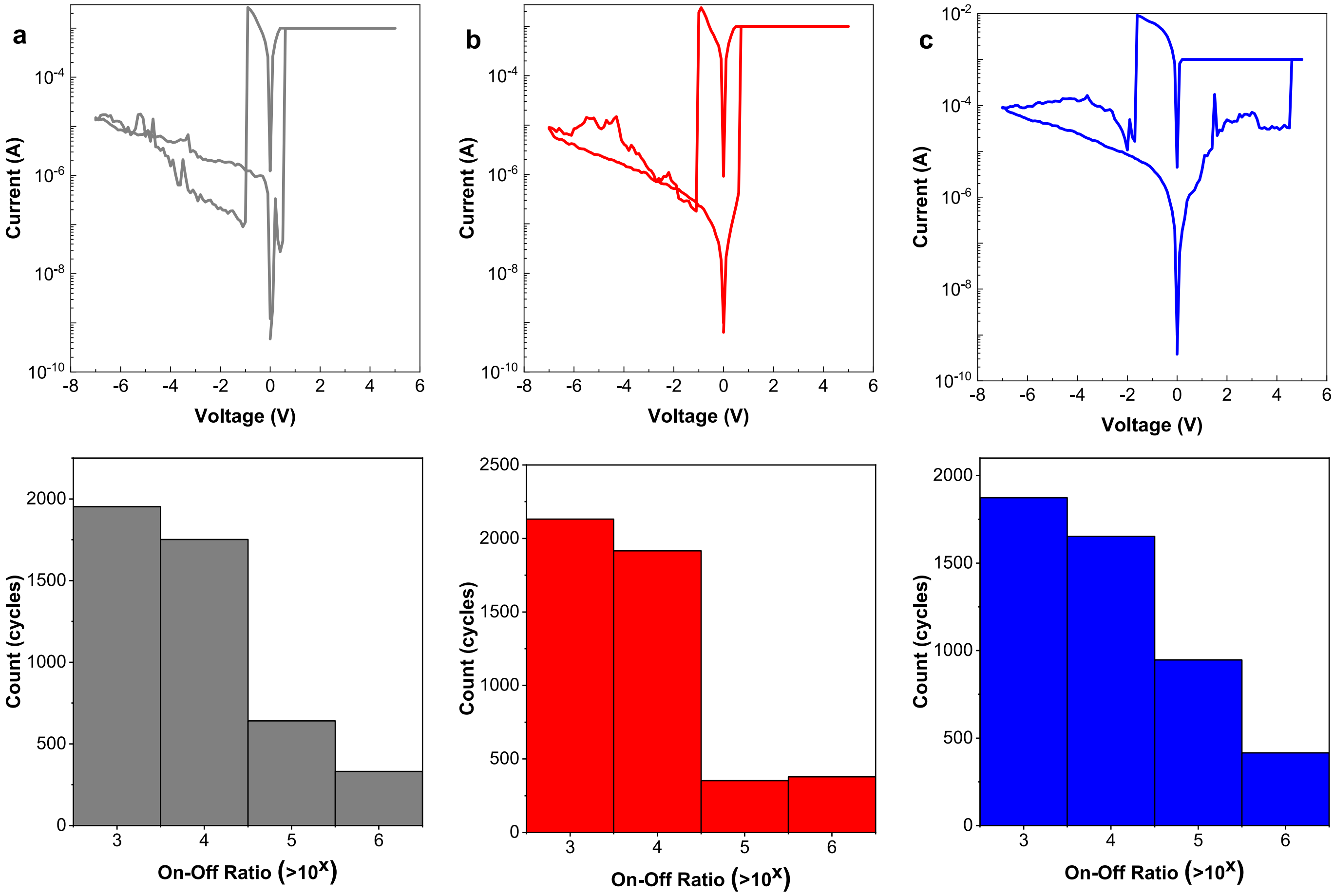

Supplementary Fig. 21. Effect of NC size. IV characteristics (top) and a representative statistical analysis of the distribution of on-off ratios during endurance testing (bottom) of **a** 13nm, **b** 9nm and **c** 7nm OGB-capped $CsPbBr_3$ NC memristors.

Supplementary Note 3: Unravelling features of short-term dynamics of diffusive perovskite memristors- enablers for reservoir computing

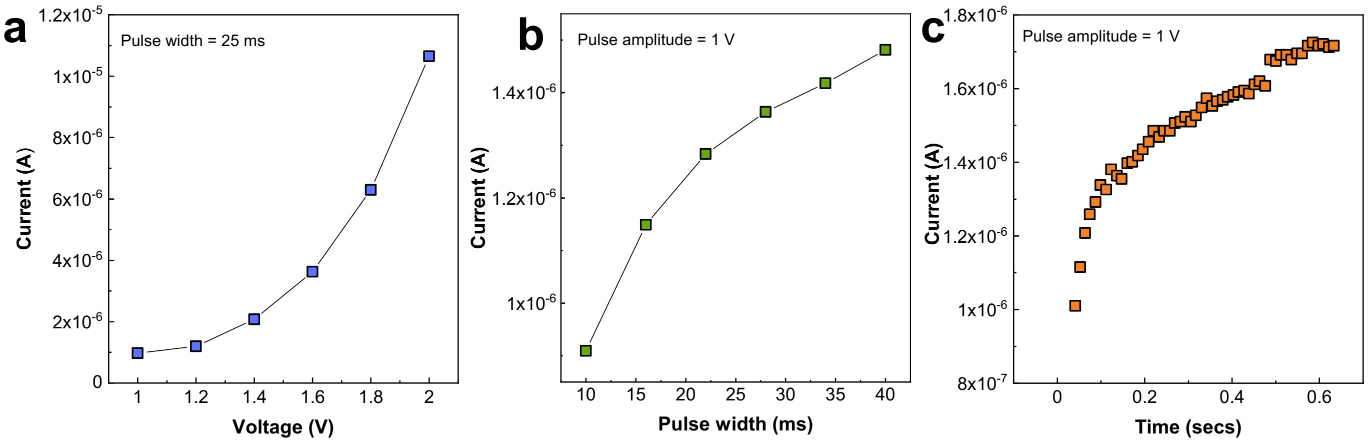

Supplementary Fig. 22. Non-linear variation of the device conductance as a function of the stimulation pulse **a** amplitude, **b** width and **c** number. For **a**, the pulse width and number is kept constant at 25 ms and 1 respectively. For **b**, the pulse amplitude and number is kept constant at 1 V and 1 respectively. For **c**, the pulse amplitude and width is kept constant at 1 V and 25 ms respectively.

The echo state property of a reservoir refers to the impact that previous inputs have on the current reservoir state, and how that influence fades out with time. To test this, four short pulses of 1 V, 5 ms are applied to the device in a paired-pulse format and the device states are recorded. A non-linear accumulative behaviour is observed as a function of the paired-pulse interval. In Supplementary Fig. 23a, a short paired-pulse interval of 10 ms results in an echo index (defined as $\left(\frac{a4}{a1}\right) * 100$) of 118 %. Longer intervals (30 ms and 300 ms in Supplementary Fig. 23b and c) result in smaller echo indices (107.5 % and 107.2 % respectively), reflective of the short-term memory in the perovskite memristors. To further test the echo state property, three pulse trains consisting of 10 identical stimulation pulses (1 V, 5 ms) are applied to the device and the device states are recorded. In all cases, a non-linear accumulative behaviour is observed. As shown in Supplementary Fig. 23d-g, short intervals ($\leq$ 23 ms) for the last stimulation pulse result in further accumulation while long intervals result in depression of the device state. This indicates that the present device state remembers the input temporal features in the recent past but not the far past, allowing the diffusive perovskite memristors to act as efficient reservoir elements. Supplementary Fig. 24 shows the evolution of the device conductance when subjected to different pulse streams. The final device conductance states show clear separation, reflecting the ability to differentiate inputs with different temporal features using distinct reservoir responses. The experimental results discussed in Supplementary Figs. 22-24 point to the short-term diffusive dynamics of the OGB-capped $CsPbBr_3$ NC memristors – key features for a reservoir layer.

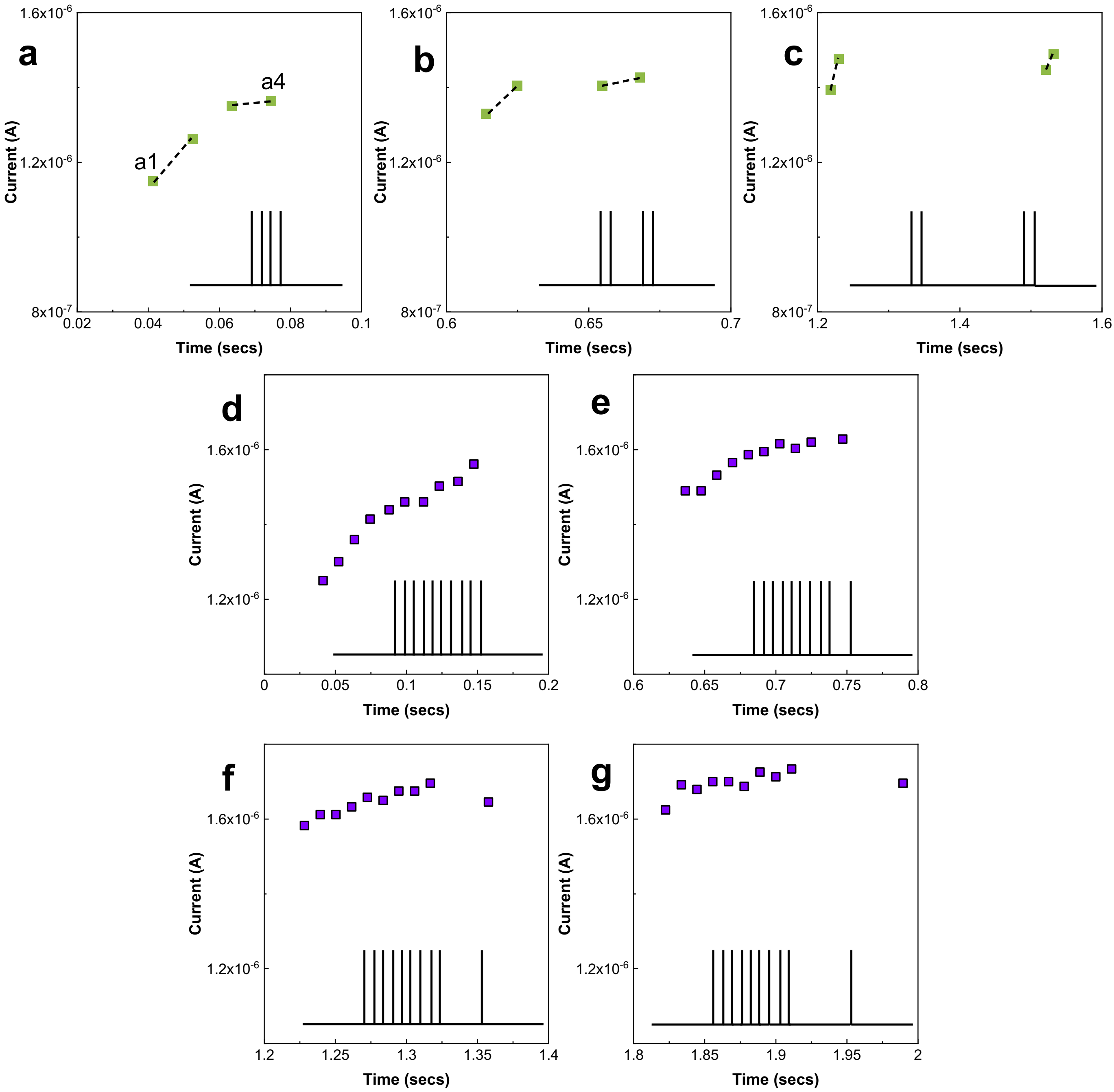

Supplementary Fig. 23. Echo state Properties. Variation in the device conductance of the volatile diffusive perovskite memristor as a function of the inter-group pulse interval. The interval between the two sequences increases from **a** 10 ms, **b** 30 ms to **c** 300 ms. **d-g** Current responses when subjected to 10 identical stimulation pulses (1 V, 5 ms) with different pulse interval conditions for the final pulse. The interval varies from **d** 10 ms, **e** 23 ms, **f** 41 ms, to **g** 80 ms.

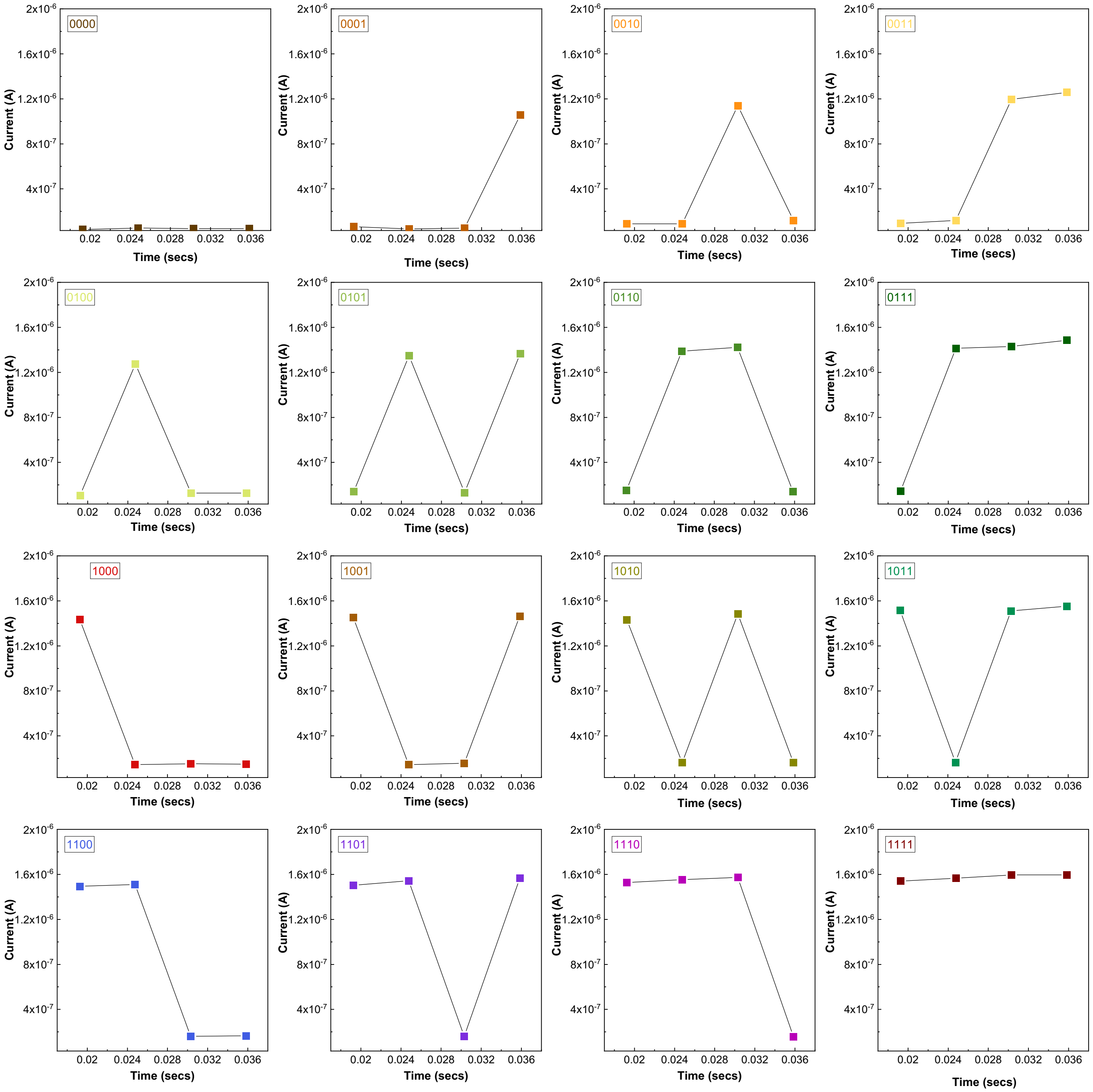

Supplementary Fig. 24. Separability for pattern encoding. Evolution of the device conductance when subjected to different pulse streams. In these experiments, state “1” is encoded by a pulse voltage of + 1 V, width = 5 ms and state “0” is represented by absence of external bias for 5ms.

Supplementary Note 4: Training neural networks with volatile and non-volatile memristive properties

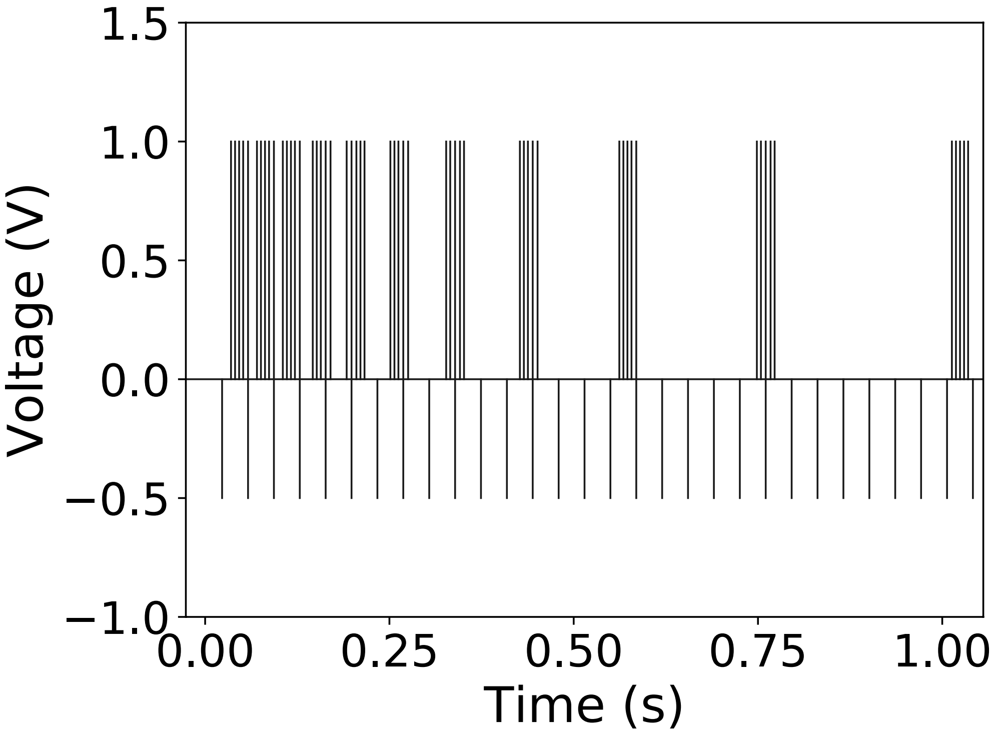

Supplementary Fig. 25. Input waveforms. A representative "Write" (amplitude = 1 V, pulse width = 20 ms) and "Read" (amplitude = - 0.5 V, pulse width = 5 ms) spike train applied to the volatile perovskite memristors in the reservoir layer.

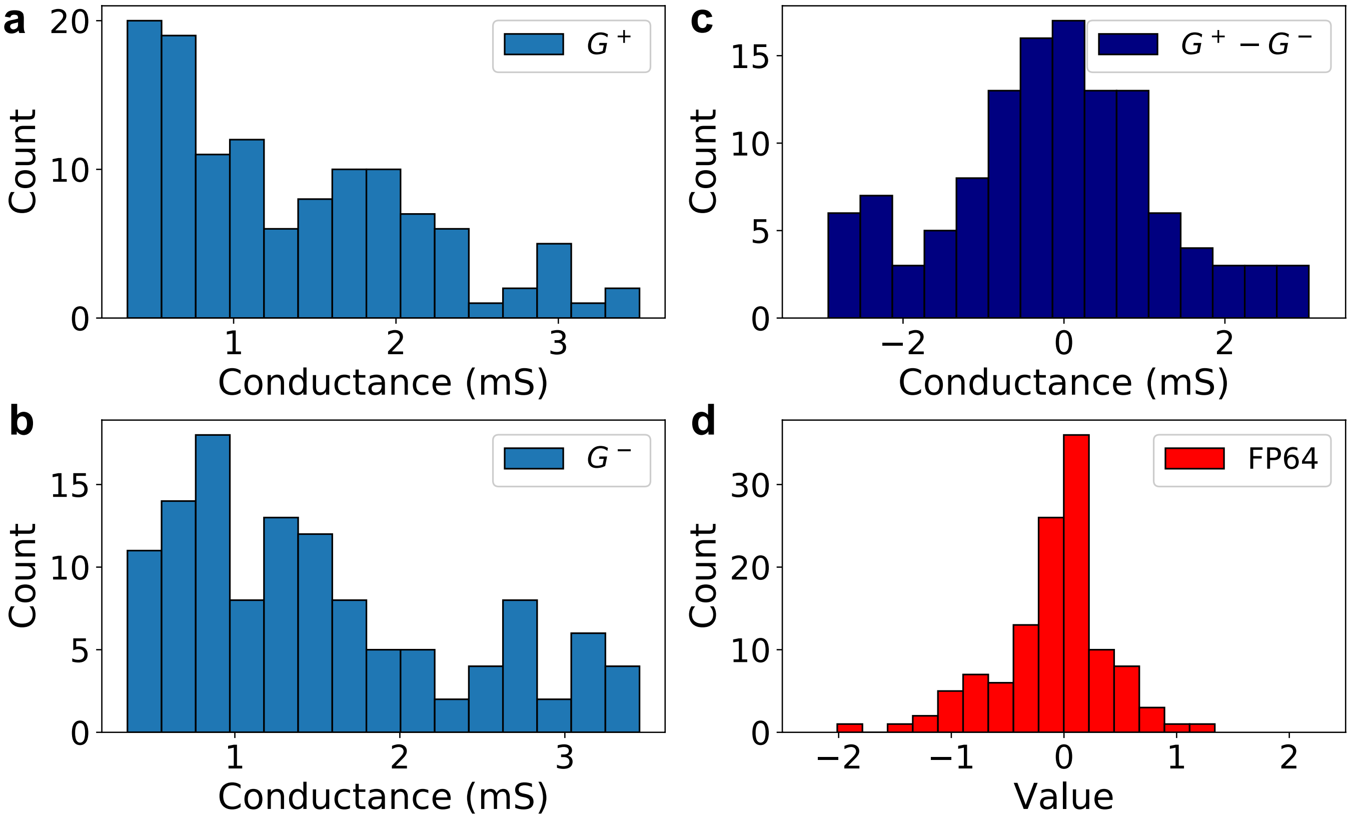

Supplementary Fig. 26. Weight distribution. The synaptic weight distribution after training. **a-b** Conductance distribution of both positive and negative differential perovskite memristors. **c** The effective

(unscaled) conductance distribution. **d** Weight distribution of the readout layer with floating-point-double precision weights. The effective memristive weights and FP64 weights follow a similar distribution.

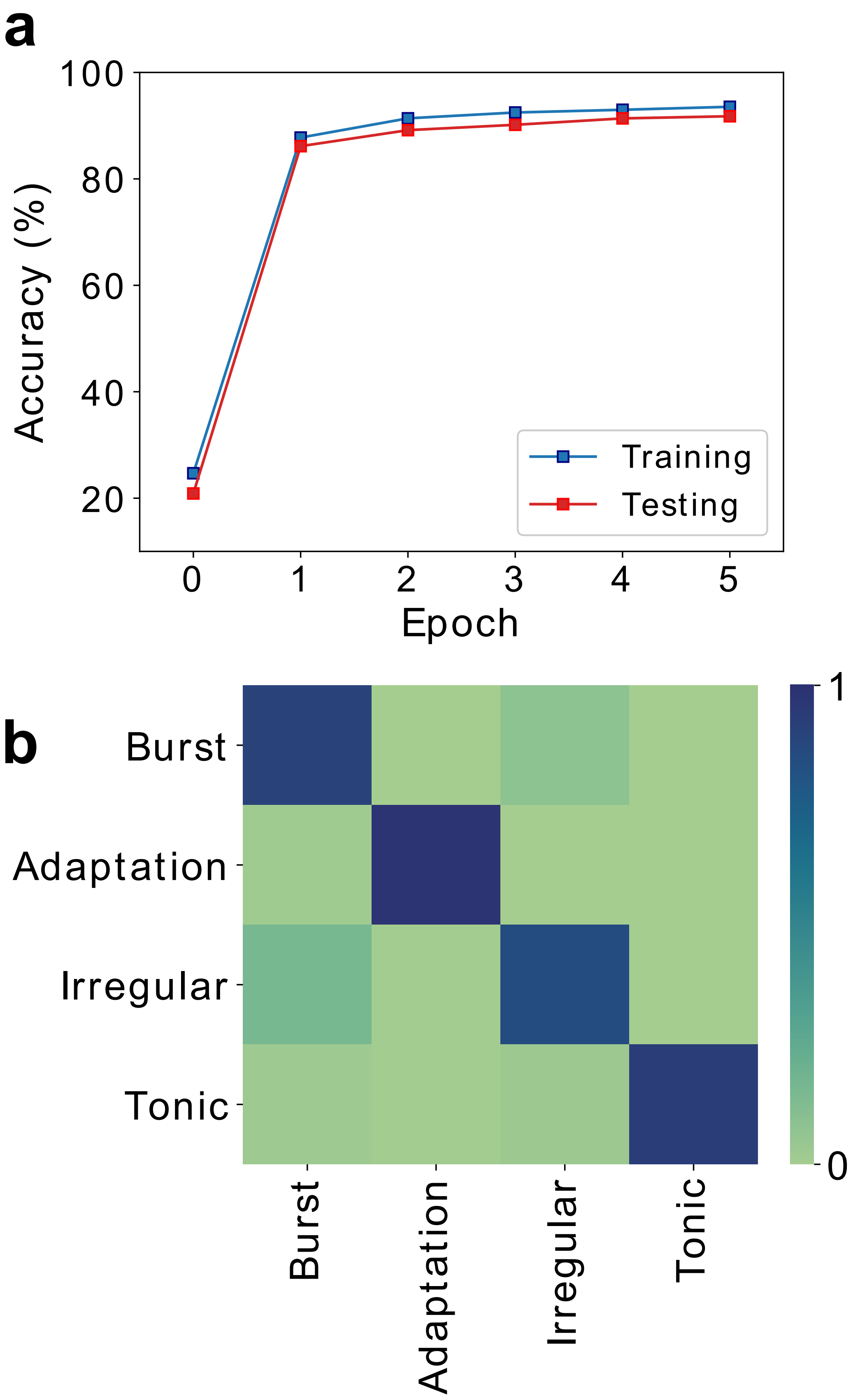

Supplementary Fig. 27. Training RC with FP64 readout weights using backpropagation. The training metrics of ANN is shown. **a** Training and testing accuracy over 5 epochs demonstrates that the network

solves the classification task with high accuracy without overfitting. **b** Confidence matrix calculated at the end of training. The correct response probability is shown in the right color scale. It is evident that network performs slightly worse in discriminating irregular patterns.

Supplementary Table 1. Comparing training and test accuracies of both approaches. The neural spiking pattern classification performance table comparing the two approaches. The readout layer with drift-based halide-perovskite memristor weights trained with online I_{cc} control achieves comparable result with FP64 weights trained with backpropagation.

		Accuracy (%)					
		Epoch 0	Epoch 1	Epoch 2	Epoch 3	Epoch 4	Epoch 5
FP64 Weights with Backpropagation	Training	24.68	87.76	91.38	92.47	92.99	93.54
	Testing	20.88	86.14	89.16	90.16	91.37	91.77
Perovskite Weights with I_{cc} Control	Training	10.32	69.11	89.12	83.09	85.59	86.75
	Testing	14.46	73.29	86.14	80.92	84.94	85.14

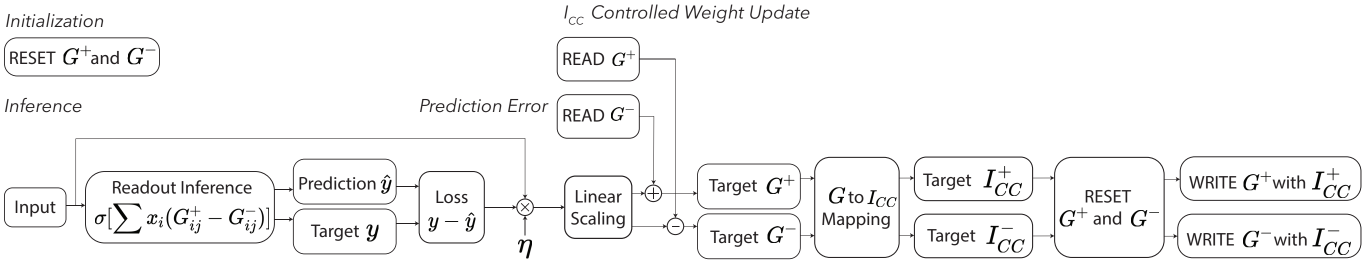

Supplementary Fig. 28. I_{cc} modulated training for drift-based perovskite configuration.

Before the training, we assumed G^+ and G^- memristors of the readout layer are initialized with RESET, followed by an iterative SET operation, resulting in random conductances in LRS with $N(0.5, 0.1)$ mS.

During the inference procedure, reservoir output vector of length 30 is fed into the readout layer. Memristors in the readout layer are placed in the differential architecture, in which the difference of conductance values of two differential memristors (G^+ and G^-) determines the effective synaptic strength. Scaled with $\beta = 1/(G_{max} - G_{min})$, where G_{max}= 0.35 mS and G_{min}=0.1 mS, the weight matrix (30 x 4) is calculated as $W = \beta[G^+ - G^-]$. And the network prediction is determined by choosing output neuron index with the maximum activation level.

For the training procedure, the network loss is calculated as the difference between output layer prediction and the one-hot encoded target vector indicating one of the four firing patterns. At this point, one can calculate targeted weights using the backpropagation algorithm. However, to support fully online-learning, we tested $\mathrm{I_{cc}}$ controlled weight update scheme where following stages in the pipeline can be easily implemented with the mixed-signal circuits in an event-driven manner. The $\mathrm{I_{cc}}$ controlled weight update is implemented as follows. First, the required weight change is calculated with $W_{target} = \eta x_i \delta_j$, where η is suitably low learning rate, x_i is the reservoir layer output and δ_j is the calculated error. In order to calculate the target conductance values for both positive and negative memristors, we first linearly scale the weight change to conductance change (by multiplying with $1/\beta$). Secondly, we read both the positive and negative conductance values. By using a push-pull mechanism, we calculate the target conductance values. The push-pull mechanism ensures a higher dynamic range in the differential configuration. Third, the target conductances are linearly mapped to the target $\mathrm{I_{cc}}$ values ($\mathrm{I}_{cc,target} = (G_{target} + 1.249 \times 10^{-5}) / 3.338$) for positive and negative memristors. The weights are updated with the application of RESET and SET pulses with the targeted $\mathrm{I_{cc}}$. Using the linear relation between $\mathrm{I_{cc}} \rightarrow G$ control, we calculated mean ($\mu_G = 3.338\,\mathrm{I_{cc}} - 1.294 \times 10^{-5}$) and standard deviation $\sigma_G = 7.040\,\mathrm{I_{cc}} + 3.0585$ and sample from the corresponding Normal distribution.

Supplementary Note 5: Comparison with state-of-the-art halide perovskite memristors

Supplementary Table 2 shows a detailed comparison against dual functional memristors (same device that exhibits both volatile and non-volatile switching) reported in literature.

Composition	Reconfigurable?	VM I_{On}/I_{Off}	VM Endurance (cycles)	NVM I_{On}/I_{Off}	NVM Retention (seconds)	NVM Endurance (cycles)	Reference
Au/Ti/h-BN/Cu	No	$10^{3\text{-}4}$	500	10^2	10^3	100	[6]
Ag-WSe_2-Ag	No	< 10^2	90	10^3	10^4	N.R.	[8]
ITO/amorphous TiO_x/Ag nanoparticles/polycrystalline TiO_x/FTO	No	10^2	10	10^2	10^4	100	[19]
Ti/Al/Ni/Au/epitaxially regrown GaN-on-GaN vertical thin films/ Ti/Al/Ni/Au/	No	10^3	10^3	10^1	N.R.	N.R.	[20]
Al/$RbPbBr_3$/ITO/PET	No	$10^{3\text{-}4}$	N.R.	$10^{3\text{-}4}$	10^4	500	[21]

Au/Ti/CuO-NWs/ Au/Ti	No	10^3	10^5	10^5	N.R.	N.R.	22
Ag/SiO_2/Pt	No	10^3	N.R.	> 10^4	N.R.	N.R.	23
Ti/*h*-BN/Cu	No	10^4	N.R.	10^4	N.R.	600	24
Ag/CFO/Pt	No	10^2	100	> 10^3	10^3	500	25
Ag/fibroin/Au configuration	Potentially Yes	10^2	10	10^6	10^4	10	26
Ag/ IGZO/MnO/Pt	Potentially Yes	10^4	N.R.	10^7	10^4	200	27
Ag/OGB capped $CsPbBr_3$ NCs/pTPD/PEDOT :PSS/ITO	Yes	10^3	2 x 10^6	$\geq 10^3$	10^5	5.6 x 10^3	This work

Key: N.R.- Not Reported

This feature of dual functionality is rare without additional materials engineering such as reported in the following works.

1. In Nature Materials 16.1 (2017): 101-108[28], a SiO_xN_y:Ag diffusive memristor was connected in series with the TaO_x drift memristor to implement a neuron and a synapse. With our reconfigurable $CsPbBr_3$ NC memristors, it becomes possible to implement both neurons and synapses with the same material/device platform.

2. Journal of Materials Chemistry C 7.39 (2019): 12160-12169[29]: Here, the electrodes were changed from Au to Ag to achieve the transition from non-volatile memory to volatile threshold switching with MoO_3 nanobelts as the active material. In our case, the same material stack is used to implement both volatile and non-volatile switching.

3. Nanoscale Advances 1.9 (2019): 3753-3760[30]: Here a Ag/HfO_x/Pt selector was transformed into a quantum-contact originated memory by introducing a graphene interface barrier layer. We do not use any form of additional materials/device engineering to implement dual functionality.

4. Scientific reports 2.1 (2012): 1-6[31]: In strongly correlated electron systems such as NiO, conversion between the non-volatile memory switching and volatile threshold switching was achieved by rationally adjusting the stoichiometry and the associated defect characteristics. In our case, the material stack remains unchanged to implement both volatile and non-volatile switching.

Thus, it is clear that a memristor that can be reconfigured to achieve on-demand switching between diffusive/volatile and drift/non-volatile modes is a research gap, that we fill with this work.

To demonstrate “reconfigurability” on-the-fly, our devices are switched between volatile and non-volatile modes with precise compliance current (I_{cc}) control and selection of activation voltages. Supplementary Fig. 29. shows that our devices can act as a volatile memory even after setting it to multiple non-volatile

states. This proves true "reconfigurability" of our devices, hitherto undemonstrated. Such behaviour is an example of the neuromorphic implementation of synapses in Spiking Neural Networks (SNNs) that demand both volatile and non-volatile switching properties, simultaneously (see Fig. 1a).

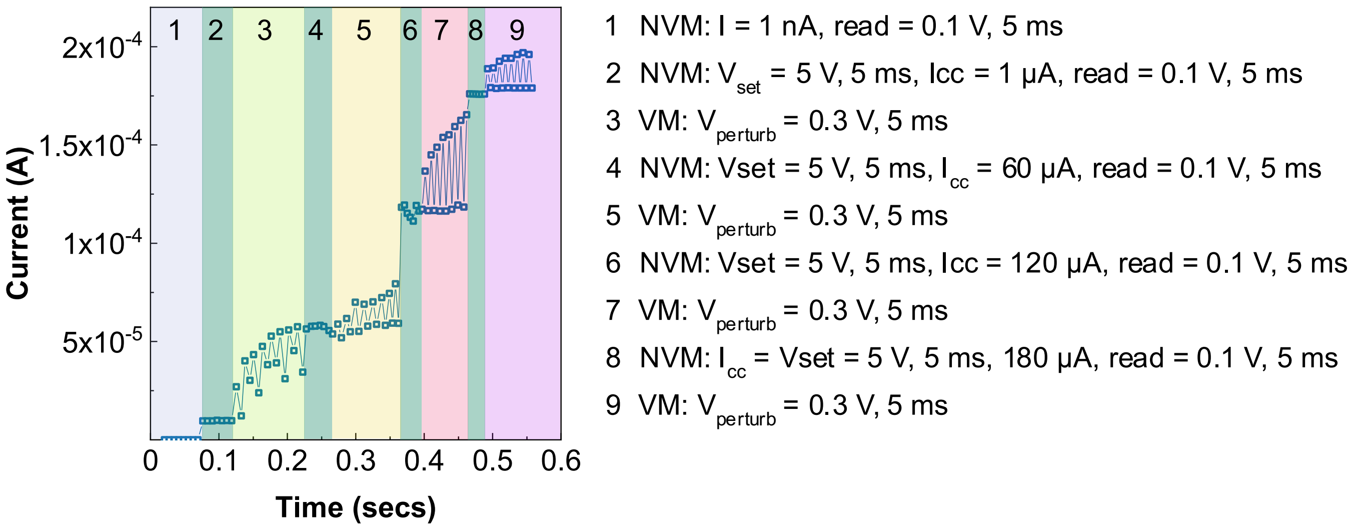

Supplementary Fig. 29. "Reconfigurability" on-the-fly of OGB-capped $CsPbBr_3$ NC memristors. The device is switched between its non-volatile and volatile mode on demand.

Supplementary Note 6: Thermal Camera Imaging

The operation of our perovskite devices depend on the migration of electrochemically active Ag^+ species through the perovskite matrix as explained in the manuscript. Here, the halide perovskite acts like a scaffold to enable this process. The formation and rupture of such conductive filaments (CFs) depend on a lot of factors. Among these factors, the applied electric field and Joule heating play the most important roles. Both these factors can play complimentary/competing roles and in determine the nature of the set/reset processes[32,33].

By setting compliance currents (I_{cc}), we essentially set a limit to the extent of electrochemical reactions that occur during the migration of Ag^+ species through the perovskite matrix, in turn controlling the thickness of the CF or number of CFs to some extent. When the device is operated under a low I_{cc} of 1μA, the filaments formed are thin and unstable and can dissolve spontaneously, resulting in a volatile memory. And since these processes have a low electrochemical and thermal budget, it becomes feasible to repeat the processes many times, resulting in high volatile endurance[34]. When the I_{cc} is raised to 1mA, the filaments formed are relatively thicker or more number of filaments can be formed, making it difficult to initiate the dissolution process. Hence, the devices preserve the CFs even when powered off, i.e. they are non-volatile. A large negative voltage is required to reset our bipolar devices and the Joule heating generated during this

process ruptures the CFs. In the non-volatile mode, since these processes have a high electrochemical and thermal budget, it becomes difficult to repeat the processes many times, resulting in low non-volatile endurance[35]. This is a common observation in all memristive devices, irrespective of the active switching material as can be seen from Supplementary Note 5 Supplementary Table 2.

To investigate this further, we monitor the working of OGB memristors in their volatile and non-volatile modes *in operando* using a thermal camera. We observe that maximum heat is generated in our memristors during the reset process when trying to break the conductive filament(s). Application of reverse bias causes Joule heating which ruptures the conductive filament(s). Thick filaments are difficult to break and result in large rise in temperature in the surrounding areas which we pick up as an infrared image[36–38]. Supplementary Fig. 30 shows the thermograms recorded at the endurance limit of the respective modes – 2 million cycles for the volatile and 5000 cycles for the non-volatile mode. When the OGB device reaches its maximum non-volatile endurance/near failure (5000 cycles), an 84°C rise in surface temperature is noted. These findings indicate that an applied field alone cannot rupture the conductive filaments and that the reset process is likely to be a combined effect of electric field and Joule heating. However, no rise in surface temperature is observable after 2 million cycles of volatile endurance, supporting our hypothesis of better management of the electrochemical reactions with lower I_{cc}. As a result, the volatile endurance is much larger when compared to the non-volatile endurance.

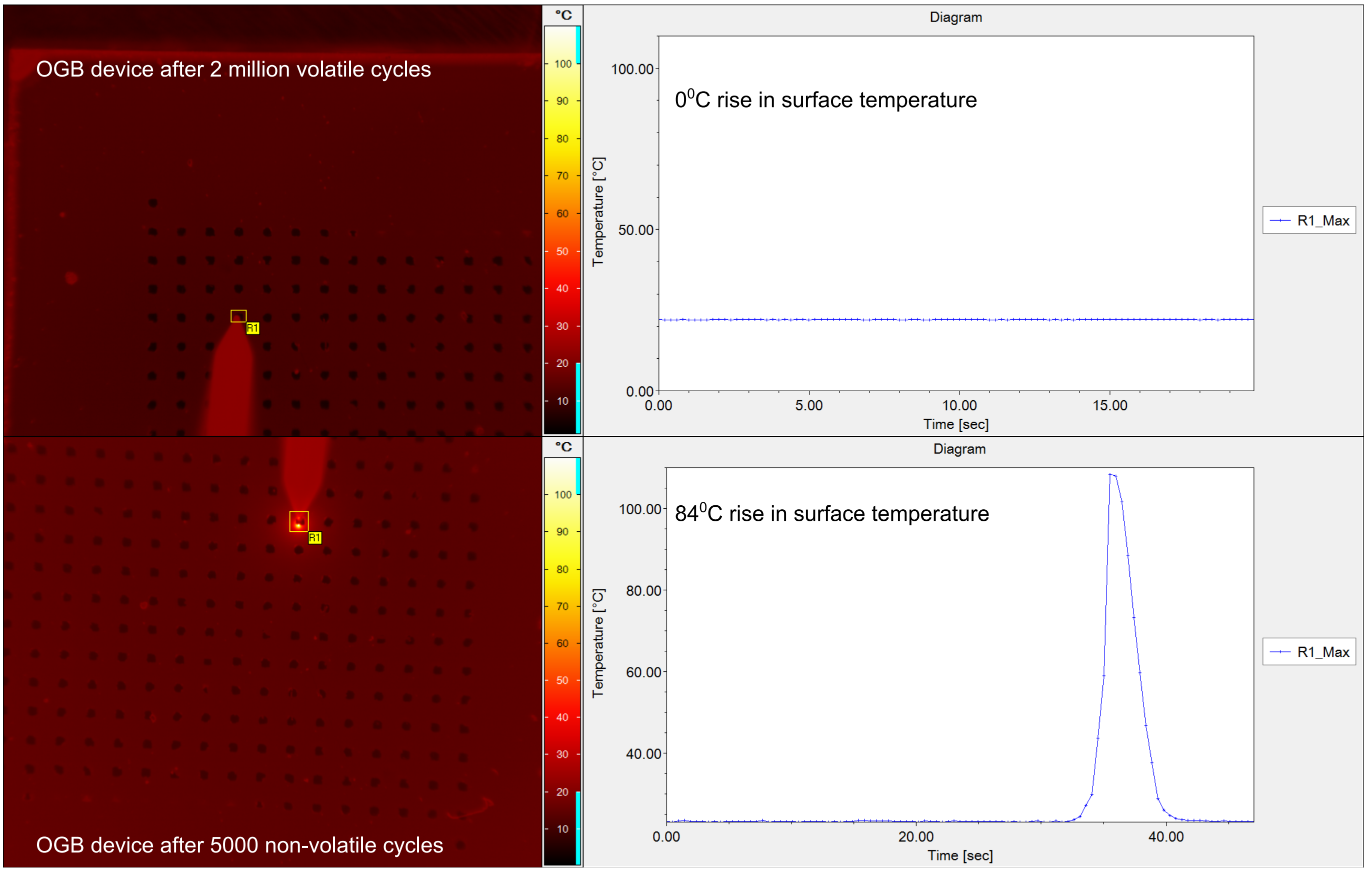

Supplementary Fig. 30. *In operando* monitoring of OGB memristors in their volatile and non-volatile modes using an infrared camera.

The dynamic ligand binding in halide perovskite (HP) nanocrystals (NCs) have been widely observed to create surface traps[39]. Therefore, handling the surface ligands without sacrificing the optoelectronic properties or affecting the structural integrity poses a challenge to developing semiconductive HP NC films. The large PL quenching observed with DDAB ligands (solution vs thin film comparison shown in Supplementary Fig. 12) point to creation of large number of surface traps. In perovskite films and NCs, it has been firmly established that trap states at the grain boundaries and on the surface can capture photo-excited charge carriers to create a local electric field capable of promoting ion migration[40–45]. In our case, this could lead to enhanced Ag^+ and Br^- migration and eventually lead to thicker Ag filament formation. From our IV endurance measurements, the DDAB devices are observed to

(i) quickly transit from a volatile to a non-volatile state, even at a low compliance current (I_{cc}) of 1 μA resulting in an inferior volatile endurance of ~ 10 cycles, and

(ii) quickly transit to a non-erasable non-volatile state at high I_{cc} of 1 mA, resulting in an inferior non-volatile endurance of ~ 50 cycles.

Both these results support the hypothesis of enhanced electrochemical reactions in the DDAB system due to their short chains.

To investigate this further, we monitor the working of DDAB and OGB memristors in their non-volatile modes *in operando* using a thermal camera. Supplementary Fig. 31 shows the thermograms recorded at the time of failure of the memristors in their non-volatile mode- 50 cycles for DDAB and 5655 cycles for OGB. For the OGB device, a thermogram is also recorded at the 50th endurance cycle for direct comparison to the DDAB device. At the time of failure of the DDAB device, i.e. 50th endurance cycle, a 95°C rise in surface temperature is seen. In comparison, only a 13°C rise is observed for OGB devices at the 50th cycle. When the OGB device reaches its maximum endurance/near failure (5655 cycles), a 86°C rise in surface temperature is noted similar to the DDAB device. These findings indicate that the reset process is likely to be a combined effect of electric field and Joule heating. Hence it is critical to engineer materials to regulate the underlying electrochemical reactions. This experiment further supports our hypothesis of better management of the electrochemical reactions with the OGB ligands when compared to DDAB, and points to the importance of investigating nanocrystal-ligand chemistry for the development of high-performance robust memristors.

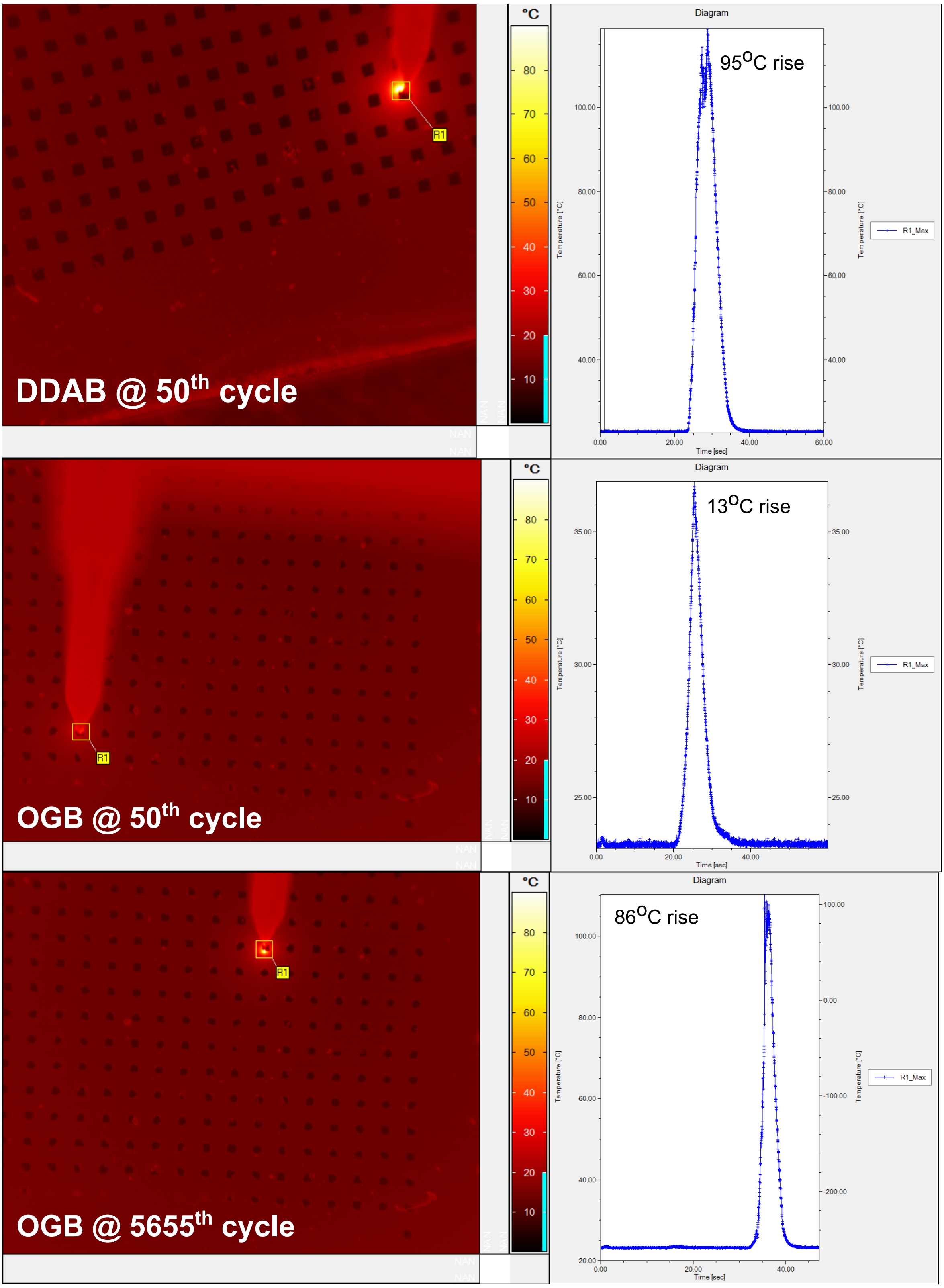

Supplementary Fig. 31. ***In operando*** **monitoring of the DDAB and OGB memristors** in their non-volatile mode using an infrared camera.

Supplementary Note 7: Thin film vs NC film

To compare thin film and NC film of $CsPbBr_3$, the thickness of the NC layer is increased to ~100nm by 5-6 serial spin coating steps and 100 nm thick $CsPbBr_3$ thin films were spin coated (5000 rpm for 30 s) from equimolar precursor solutions of 0.25 M CsBr and 0.25 M $PbBr_2$ in DMSO solvent and annealed at 100 °C for 15 min under nitrogen environment to remove the solvent residue. The thicknesses of the other interlayers were fixed to: PEDOT:PSS- 30nm, pTPD- 20nm, and Ag- 150nm.

Both configurations exhibit very similar characteristics with an on-off ratio $\geq 10^3$, similar set and reset voltages. The volatile endurance performance is also similar and lasts 2 million cycles in both the thin film and NC film format (data not shown). However, stress tests of endurance and retention in the non-volatile mode reveal distinct responses. While $CsPbBr_3$ NCs depict high retention (10^5 secs) and endurance (5655 cycles) performance in the non-volatile mode, $CsPbBr_3$ thin films portray low retention (10^4 secs) and endurance (1174 cycles) performance in the non-volatile mode (Supplementary Fig. 32). These results point to different migration pathways for the Ag^+ ion in NCs vs thin films.

Most importantly, we observe that $CsPbBr_3$ thin film memristors fail to be reconfigured back to their volatile mode once their non-volatile mode is activated, similar to other dual functional memristors reported in literature. On the other hand, the NC-based memristor allow facile reconfiguration between the volatile and non-volatile modes (Supplementary Fig. 33).

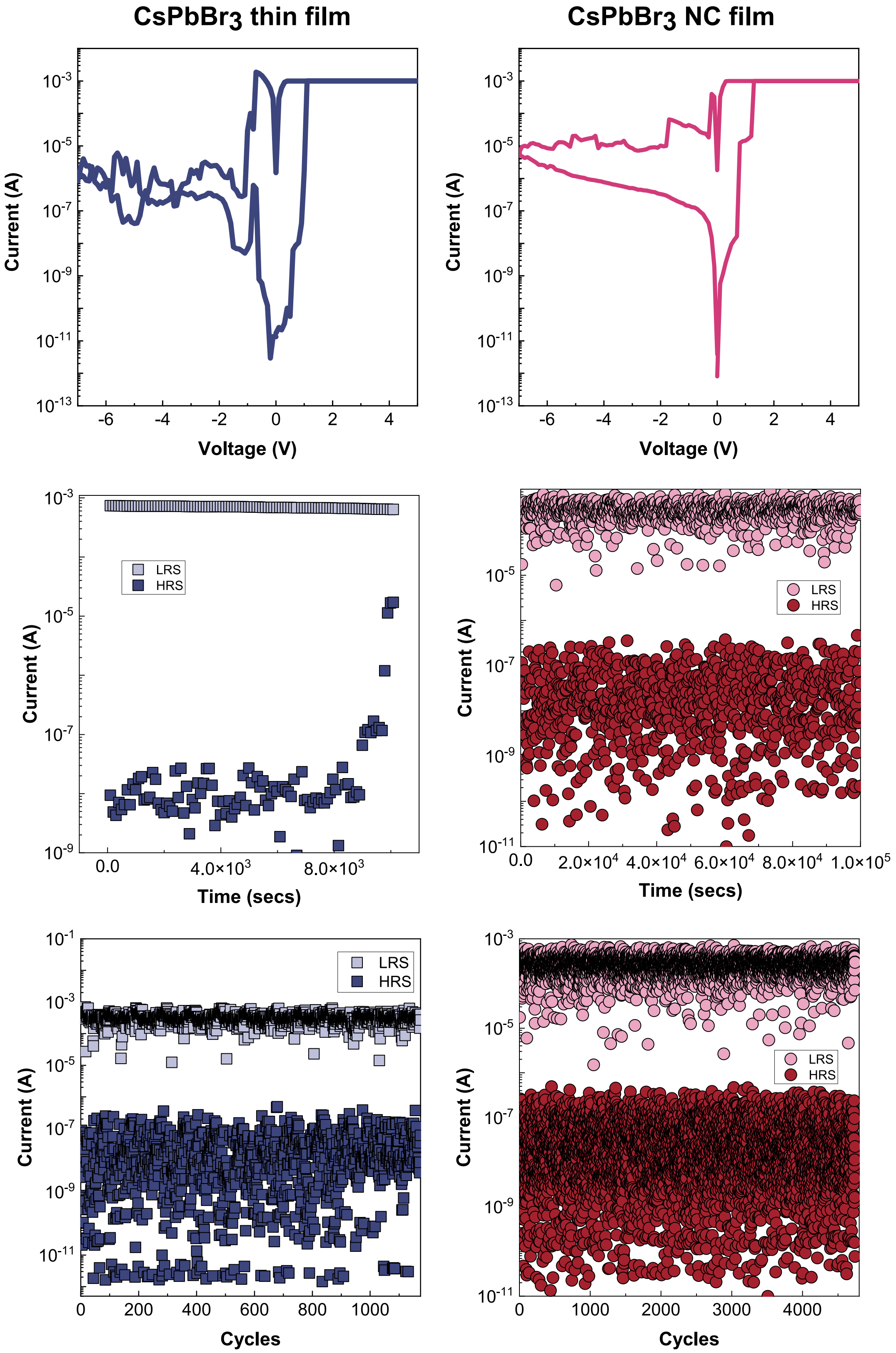

Supplementary Fig. 32. Thin film vs NC film comparison. (reading top to bottom) I–V characteristics, retention and endurance performance of $CsPbBr_3$ thin film (left) and NC film (right) memristor in their non-volatile mode.

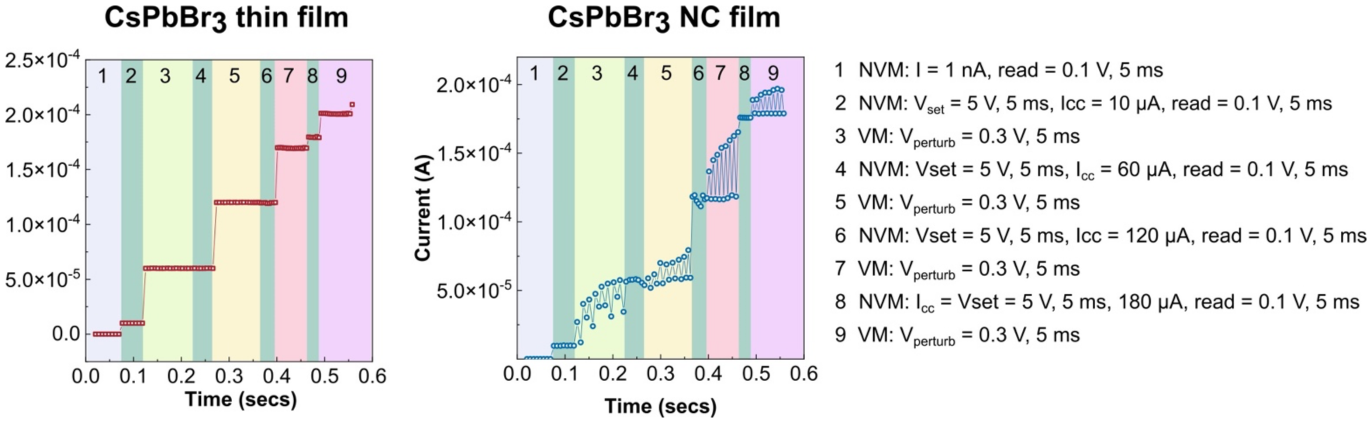

Supplementary Fig. 33. Switching between volatile and non-volatile modes on demand. $CsPbBr_3$ thin film memristors are unable to switch to a volatile mode once the non-volatile mode is activated, but the OGB-capped $CsPbBr_3$ NC memristors can facilely switch between the 2 modes.

While the exact memristive mechanism is still unclear, our results favour NC memristor implementations empirically. Theoretically, it is plausible for a single nanocrystal to support multiple Ag filament formation-disruption processes. Since a single device encompasses 1000s of such nanocrystals, the NC layer provides a pool of electrochemically-active sites for filament formation. In comparison to thin films, where a specific migration path along grain boundaries maybe preferentially opted, the NC layer provides larger number of possibilities for forming CFs. Moreover, the ligands can further control the extent of electrochemical reactions as seen from our *in operando* thermal imaging experiments. Our work shows that larger insulating ligands may fair better in controlling the electrochemical reactions, hence resulting in higher endurance. But this is only a preliminary study and much more needs to be unravelled in future works, and advances in halide perovskite nanocrystal chemistry will play a significant role in this regard.

Supplementary References:

1. Bodnarchuk, M. I. *et al.* Rationalizing and controlling the surface structure and electronic passivation of cesium lead halide nanocrystals. *ACS energy Lett.* **4**, 63–74 (2018).
2. Lee, S. *et al.* Tailored 2D/3D Halide Perovskite Heterointerface for Substantially Enhanced Endurance in Conducting Bridge Resistive Switching Memory. *ACS Appl. Mater. Interfaces* **12**, 17039–17045 (2020).
3. John, R. A. *et al.* Ionotronic Halide Perovskite Drift-Diffusive Synapses for Low-Power Neuromorphic Computation. *Adv. Mater.* 1805454 (2018).
4. Zhu, X., Lee, J. & Lu, W. D. Iodine vacancy redistribution in organic–inorganic halide perovskite films and resistive switching effects. *Adv. Mater.* **29**, 1700527 (2017).
5. Nedelcu, G. *et al.* Fast anion-exchange in highly luminescent nanocrystals of cesium lead halide perovskites (CsPbX3, X= Cl, Br, I). *Nano Lett.* **15**, 5635–5640 (2015).
6. Shi, Y. *et al.* Electronic synapses made of layered two-dimensional materials. *Nat. Electron.* **1**,

458–465 (2018).
7. Xiao, N. *et al.* Resistive random access memory cells with a bilayer TiO2/SiOX insulating stack for simultaneous filamentary and distributed resistive switching. *Adv. Funct. Mater.* **27**, 1700384 (2017).
8. Sivan, M. *et al.* All WSe 2 1T1R resistive RAM cell for future monolithic 3D embedded memory integration. *Nat. Commun.* **10**, 1–12 (2019).
9. Kato, Y. *et al.* Silver iodide formation in methyl ammonium lead iodide perovskite solar cells with silver top electrodes. *Adv. Mater. Interfaces* **2**, 1500195 (2015).
10. Svanstrom, S. *et al.* Degradation mechanism of silver metal deposited on lead halide perovskites. *ACS Appl. Mater. Interfaces* **12**, 7212–7221 (2020).
11. Zhang, T. *et al.* Profiling the organic cation-dependent degradation of organolead halide perovskite solar cells. *J. Mater. Chem. A* **5**, 1103–1111 (2017).
12. Safari, Z. *et al.* Optimizing the interface between hole transporting material and nanocomposite for highly efficient perovskite solar cells. *Nanomaterials* **9**, 1627 (2019).
13. Hofmann, S. Sputter depth profile analysis of interfaces. *Reports Prog. Phys.* **61**, 827 (1998).
14. Vriezema, C. J. & Zalm, P. C. Impurity migration during SIMS depth profiling. *Surf. interface Anal.* **17**, 875–887 (1991).
15. Ilyas, N. *et al.* Analog switching and artificial synaptic behavior of Ag/SiO x: Ag/TiO x/p++-Si memristor device. *Nanoscale Res. Lett.* **15**, 1–11 (2020).
16. Yoo, E. *et al.* Bifunctional resistive switching behavior in an organolead halide perovskite based Ag/CH 3 NH 3 PbI 3− x Cl x/FTO structure. *J. Mater. Chem. C* **4**, 7824–7830 (2016).
17. Duijnstee, E. A. *et al.* Toward understanding space-charge limited current measurements on metal halide perovskites. *ACS Energy Lett.* **5**, 376–384 (2020).
18. Le Corre, V. M. *et al.* Revealing Charge Carrier Mobility and Defect Densities in Metal Halide Perovskites via Space-Charge-Limited Current Measurements. *ACS energy Lett.* **6**, 1087–1094 (2021).
19. Huang, Y.-J. *et al.* Dual-functional memory and threshold resistive switching based on the push-pull mechanism of oxygen ions. *Sci. Rep.* **6**, 1–10 (2016).
20. Fu, K. *et al.* Threshold switching and memory behaviors of epitaxially regrown GaN-on-GaN vertical p-n diodes with high temperature stability. *IEEE Electron Device Lett.* **40**, 375–378 (2019).
21. Das, U. *et al.* Compliance Current-Dependent Dual-Functional Bipolar and Threshold Resistive Switching in All-Inorganic Rubidium Lead-Bromide Perovskite-Based Flexible Device. *ACS Appl. Electron. Mater.* **2**, 1343–1351 (2020).
22. Huang, C.-H., Matsuzaki, K. & Nomura, K. Threshold switching of non-stoichiometric CuO nanowire for selector application. *Appl. Phys. Lett.* **116**, 23503 (2020).

23. Sun, H. *et al.* Direct observation of conversion between threshold switching and memory switching induced by conductive filament morphology. *Adv. Funct. Mater.* **24**, 5679–5686 (2014).

24. Pan, C. *et al.* Coexistence of grain-boundaries-assisted bipolar and threshold resistive switching in multilayer hexagonal boron nitride. *Adv. Funct. Mater.* **27**, 1604811 (2017).

25. Munjal, S. & Khare, N. Compliance current controlled volatile and nonvolatile memory in Ag/CoFe2O4/Pt resistive switching device. *Nanotechnology* **32**, 185204 (2021).

26. Wang, H. *et al.* Configurable Resistive Switching between Memory and Threshold Characteristics for Protein-Based Devices. *Adv. Funct. Mater.* **25**, 3825–3831 (2015).

27. Abbas, H. *et al.* Reversible transition of volatile to non-volatile resistive switching and compliance current-dependent multistate switching in IGZO/MnO RRAM devices. *Appl. Phys. Lett.* **114**, 93503 (2019).

28. Wang, Z. *et al.* Memristors with diffusive dynamics as synaptic emulators for neuromorphic computing. *Nat. Mater.* **16**, 101–108 (2017).

29. Du, H. *et al.* Transition from nonvolatile bipolar memory switching to bidirectional threshold switching in layered MoO 3 nanobelts. *J. Mater. Chem. C* **7**, 12160–12169 (2019).

30. Wu, Z. *et al.* Transformation of threshold volatile switching to quantum point contact originated nonvolatile switching in graphene interface controlled memory devices. *Nanoscale Adv.* **1**, 3753–3760 (2019).

31. Peng, H. Y. *et al.* Deterministic conversion between memory and threshold resistive switching via tuning the strong electron correlation. *Sci. Rep.* **2**, 1–6 (2012).

32. Sun, W. *et al.* Understanding memristive switching via in situ characterization and device modeling. *Nat. Commun.* **10**, 1–13 (2019).

33. Kim, S., Kim, H. & Choi, S. Compact Two-State-Variable Second-Order Memristor Model. *small* **12**, 3320–3326 (2016).

34. Guo, M. Q. *et al.* Unidirectional threshold resistive switching in Au/NiO/Nb: SrTiO3 devices. *Appl. Phys. Lett.* **110**, 233504 (2017).

35. Chen, Y. Y. *et al.* Understanding of the endurance failure in scaled HfO 2-based 1T1R RRAM through vacancy mobility degradation. in *2012 International Electron Devices Meeting* 20–23 (IEEE, 2012).

36. Gogoi, H. J. & Mallajosyula, A. T. Enhancing the Switching Performance of CH3NH3PbI3 Memristors by the Control of Size and Characterization Parameters. *Adv. Electron. Mater.* 2100472 (2021).

37. Li, Y., Wang, Z., Midya, R., Xia, Q. & Yang, J. J. Review of memristor devices in neuromorphic computing: materials sciences and device challenges. *J. Phys. D. Appl. Phys.* **51**, 503002 (2018).

38. Yoon, K. J. *et al.* Electrically-generated memristor based on inkjet printed silver nanoparticles. *Nanoscale Adv.* **1**, 2990–2998 (2019).

39. Xue, J., Wang, R. & Yang, Y. The surface of halide perovskites from nano to bulk. *Nat. Rev. Mater.* **5**, 809–827 (2020).

40. DeQuilettes, D. W. *et al.* Photo-induced halide redistribution in organic–inorganic perovskite films. *Nat. Commun.* **7**, 1–9 (2016).

41. Azpiroz, J. M., Mosconi, E., Bisquert, J. & De Angelis, F. Defect migration in methylammonium lead iodide and its role in perovskite solar cell operation. *Energy Environ. Sci.* **8**, 2118–2127 (2015).

42. Ahn, N. *et al.* Trapped charge-driven degradation of perovskite solar cells. *Nat. Commun.* **7**, 1–9 (2016).

43. Kim, Y.-H., Wolf, C., Kim, H. & Lee, T.-W. Charge carrier recombination and ion migration in metal-halide perovskite nanoparticle films for efficient light-emitting diodes. *Nano Energy* **52**, 329–335 (2018).

44. Kamat, P. V & Kuno, M. Halide ion migration in perovskite nanocrystals and nanostructures. *Acc. Chem. Res.* **54**, 520–531 (2021).

45. Chen, M., Shan, X., Geske, T., Li, J. & Yu, Z. Manipulating ion migration for highly stable light-emitting diodes with single-crystalline organometal halide perovskite microplatelets. *ACS Nano* **11**, 6312–6318 (2017).
